# Auto-segmentation of organs-of-interest clinical acceptability & reproducibility framework in head and neck cancer

**DOI:** 10.1016/j.phro.2026.101015

**Published:** 2026-06-06

**Authors:** Joseph Marsilla, Mattea L. Welch, Joshua Siraj, Clare McElcheran, Jun Won Kim, Denis Tkachuck, Sejin Kim, John Cho, Ezra Hahn, Ali Hosni, J.C. Jacinto, Michal Kazmierski, Katrina Rey-McIntyre, Shao Hui Huang, Tirth Patel, Tony Tadic, Scott Bratman, Andrew Hope, Benjamin Haibe-Kains

**Affiliations:** a Princess Margaret Cancer Centre, University Health Network, Toronto, Ontario, Canada; b Department of Medical Biophysics, University of Toronto, Toronto, Ontario, Canada; c Department of Radiation Oncology, Gangnam Severance Hospital, Yonsei University College of Medicine, Seoul, Republic of Korea; d Department of Radiation Oncology, University of Toronto, Toronto, Ontario, Canada; e Radiation Medicine Program, Princess Margaret Cancer Center, University Health Network, Toronto, Ontario, Canada; f Department of Computer Science, University of Toronto, Toronto, Ontario, Canada; g Vector Institute, Toronto, Ontario, Canada; h Cancer Digital Intelligence Program, Princess Margaret Cancer Center, Toronto, Ontario, Canada; i Structural Genomics Consortium

**Keywords:** Auto-segmentation, Head and neck cancer, Organs-of-interest, Framework, Clinical assessment, Generalizability assessment, Reproducibility

## Abstract

**Background and purpose:**

Auto-segmentation of organs-of-interest (OOI) in cancer patients is essential for facilitating radiotherapy planning and reducing inter-observer variability. Deep learning-based auto-segmentation models have shown promise, but limited transparency and reproducibility hinder their generalizability and clinical acceptability, limiting their use in clinical settings.

**Materials and methods:**

We introduced an auto-Segmentation Clinical Acceptability & Reproducibility Framework, a comprehensive framework designed to benchmark open-source deep learning models for auto-segmentation of 19 essential OOIs in head and neck cancer (HNC). Reproducibility was achieved through harmonized data curation, standardized model training (training/tune/test of 479/44/59) and assessment workflows. New models can be benchmarked against 12 pre-trained open-source deep learning models, while estimating clinical acceptability using a 5-point Likert scale.

**Results:**

The framework codebase is openly available for benchmarking OOI auto-segmentation methods. During development, expert assessment of the best performing model labelled 16/19 AI-generated OOI categories as clinically acceptable with only minor revisions.

**Conclusions:**

The framework facilitates benchmarking and expert assessment of AI-driven auto-segmentation tools, addressing the need for transparency and reproducibility in this domain. Through its emphasis on clinical acceptability, our framework fosters the integration of AI models into clinical environments, specifically within radiation therapy.

## Highlights

• Introduced a framework for evaluating and comparing auto-segmentation models.
• Harmonized data and standardized workflows ensure full reproducibility.
• Integrated clinical acceptability assessed using expert 5-point Likert scoring.
• Benchmarked open-source auto-segmentation models on 19 Organs-of-Interest.
• 16/19 AI-generated Organs-of-Interest clinically acceptable with minor revisions.

## Graphical abstract

**Figure:**
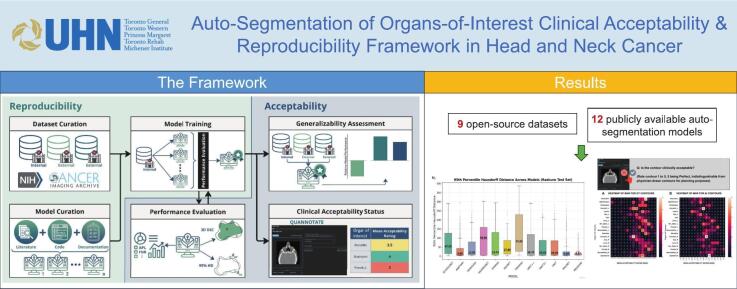

## 1 Introduction

Radiation therapy (RT) is an important cancer treatment that requires segmentation of organs-of-interest (OOI) alongside gross tumor volumes. OOI segmentation ensures RT safety and quality by optimizing tumor control and reducing radiation adverse events. Prior to AI-powered auto-segmentation, this process was manual, subjective, and time-consuming. Deep learning-based auto-segmentation has emerged as a potential solution, especially in head and neck cancer (HNC) [1], [2], [3], [4], [5], [6], [7], [8], [9], [10], [11], [12], [13].

As AI model development and adoption becomes more commonplace, numerous investigations into model evaluation [14], AI ethics [15], [16] and best practices [17] have emerged. These works provide necessary guidance on the impact of AI model adoption to patients and healthcare practice. For auto-segmentation, before clinical adoption, rigorous validation with AI-specific checklists (CONSORT-AI [18]) may be required. Although guidelines exist for reporting model performance [18], [19], [20], [21], [22], infrastructure and standardized code-bases are scarce, hindering reproducibility and critical evaluation.

Auto-segmentation model performance depends on numerous factors, including data quality and diversity, evaluation metrics, and application context [23]. Isensee et al. [22] highlighted validation pitfalls, including lack of standardized baseline models, insufficient data, and inconsistent reporting. Evaluations of auto-segmentation models often focus on geometric analysis of contour quality [24]. These measures provide comparable quantitative values, but may not represent clinical utility [20], [24]. For practical clinical implementation, additional metrics are needed to define clinical acceptability of auto-segmentation [23].

We aimed to improve validation practices, and promote reproducibility with the auto-Segmentation Clinical Acceptability and Reproducibility Framework, a modular framework adaptable to institutional resources, enabling responsible clinical deployment.

## 2 Materials and methods

### 2.1 The framework

**Fig. 1:**
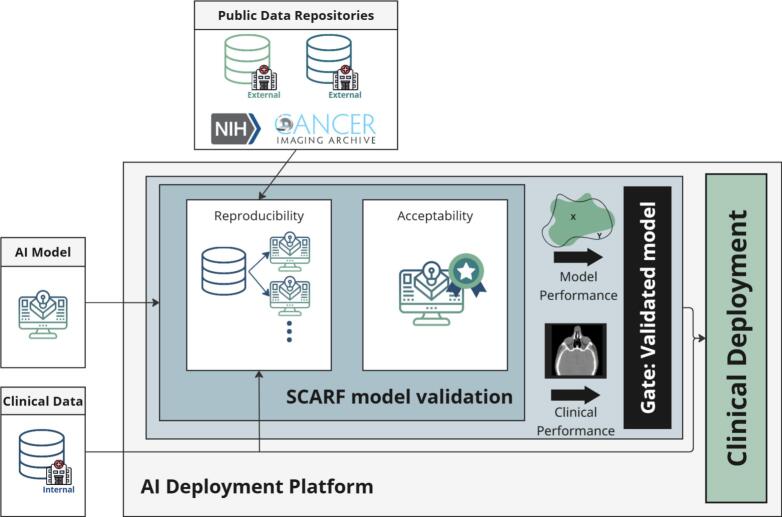
SCARF for clinical deployment. Example of SCARF embedded in MIRA clinical deployment platform (University Health Network, Toronto, Canada). A potential Model can be validated using SCARF, evaluating generalizability and clinical performance through public repository data, allowing for comparison with other models, and local clinical data. Institutions can define performance requirements for model performance and clinical performance that the model must pass to be deployed within the clinic.

The auto-Segmentation Clinical Acceptability and Reproducibility Framework (SCARF) consists of five steps (Supplementary Fig. S1): (1) Curate datasets; (2) identify relevant existing models and refine the best performing model, or, train a new auto-segmentation model for quantitative comparison; (3) quantitatively evaluate all trained models; (4) assess the clinical acceptability of best performing transparent model; and (5) test the generalizability of best performing model on additional datasets. SCARF reports median geometric performance and a numeric clinical evaluation value for each model. The framework can be integrated into a model deployment platform within an institution to evaluate potential models, and can incorporate performance thresholds to ensure high-performance models (Fig. 1). To illustrate the utility of the framework, we presented a representative case study.

### 2.2 Representative case study

#### 2.2.1 Selection of auto-segmentation models

We identified auto-segmentation models published between 2016 and 2025 with available data, code, and documentation sufficient for re-implementation (Supplementary Table S2). A literature search from January 2016 excluded 2D architectures that could not be reliably adapted to 3D. Selection criteria prioritized models originally implemented in PyTorch, designed for 3D inputs or easily modified for 3D, and favored popular repositories based on GitHub ratings with similar architectural layouts.

Selected networks were trained end-to-end with minimal changes to original source code, and refactored into a PyTorch Lightning framework [19], [21]. To isolate architectural effects on performance, all models used a consistent loss function tailored for semantic segmentation with high class imbalance [19], [25], [26], [27]: a weighted TopK Cross-Entropy loss [28] selecting the top 10% hardest voxels combined with Focal Tversky Loss.

Based on the selection and exclusion criteria outlined in our methods, we selected 12 open-source models to train on the segmentation of the 19 OOIs of interest [7], [29], [30], [31], [32], [33], [34], [35], [36], [37], [38]: 1) 3D-UNET (WOLNET) [29], [39], [40]; 2) 3D-RESUNET [30], [39], [40]; 3) HIGHRESNET [31], [41]; 4) PIPOFAN [32], [42]; 5) UNET3+ [33], [43]; 6) UNET++ [34], [43]; 7) ANATOMY [7], [44]; 8) DENSEVOX [35], [45]; 9) TIRAMISU [36], [46]; 10) RSANET [37], [47]; 11) VNET [38]; and 12) MedSAM2 [48]. Details of the model architectures can be found in Supplementary Table S2.

#### 2.2.2 SCARF 1: dataset curation

Radiological and clinical data extracted from institutional databases or public repositories, such as The Cancer Imaging Archive (TCIA) [49], often require a high level of curation in preparation for deep learning. We developed a custom script that was used in concert with Med-ImageTools [50], to curate nine radiation therapy planning computed tomography (CT) HNC datasets from nine institutions, totalling 3251 patients, ensuring no overlap between patients found in datasets (Table 1): 1) RADCURE (*n* = 2552), University Health Network [51]; 2) HNSCC-3DCT-RT (*n* = 87), MIAMI [52], [53]; 3) Deepmind (*n* = 35), HMS + Multi-Site [28]; 4) PDDCA (*n* = 48), HMS [54]; 5) Radiomics-HN1 (*n* = 129), MAASTRO [55], [56], [57]; 6) STRUCTSEG19 (*n* = 50), CAS [58]; 7) Head-Neck-CT-Atlas (*n* = 190), MDACC [59], [60]; 8) SegRap 2023 (*n* = 120), UESTC [61]; and 9) HaN-Seg (*n* = 42), Slovenia [62].

**Table 1:** External HNC imaging and segmentation datasets. All datasets are openly available for download and research purposes.

Dataset	Institution	Scans	Use in Manuscript	Available Data		
				Imaging	Clinical	OOI
RADCURE	UHN	2552	Training/Tuning/Internal Testing	☑️	☑️	☑️
HNSCC-3DCT-RT	MIAMI	94	Generalizability Assessment	☑️	☑️	☑️
Deepmind	HMS + Multi-Site	35	Generalizability Assessment	☑️		☑️
PDDCA	HMS	48	Generalizability Assessment	☑️		☑️
Radiomics-HN1	MAASTRO	137	Generalizability Assessment	☑️	☑️	☑️
STRUCT SEG19	CAS	50	Generalizability Assessment	☑️		☑️
Head-Neck-CT-Atlas	MDACC	215	Generalizability Assessment	☑️	☑️	☑️
SegRap 2023	UESTC	120	Generalizability Assessment	☑️		☑️
HaN-Seg	SLOVENIA	42	Generalizability Assessment	☑️		☑️

Med-ImageTools [50] collected each patient's CT imaging meta-data and curated ground truth radiation therapy structure files (RT-STRUCT), containing clinical contours used during RT planning. Contour names were extracted and standardized using dataset specific custom scripts. All images and contours for these datasets were generated during clinical practice and would have followed their specific institutional guidelines. These guidelines were not consistently reported for the datasets, and therefore an assumption must be made that they varied between institutions (see Supplementary Material).

Within RADCURE, a total of 34 distinct OOIs were delineated. Not all datasets contained all contours. To ensure consistent and complete supervision during training [63], only patients with delineated contours for 19 OOIs deemed essential for RT-planning were included. These 19 OOIs were: brainstem, chiasm, esophagus, acoustics (L/R), larynx, eye (L/R), lips, lens (L/R), optic nerve (L/R), parotid gland (L/R), brachial plexus (L/R), mandible, and spinal cord.

#### 2.2.3 SCARF 2: training of auto-segmentation models

Models were trained on the RADCURE dataset to segment 19 clinician-identified OOIs essential for RT planning, using a fixed training, tuning, and testing split of 479, 44, and 59 scans, respectively. All training images were resampled to 1 mm × 1 mm × 3 mm voxels for improved computational efficiency, using four NVIDIA Tesla P100 GPUs over three days or until convergence. Early stopping was triggered if tuning loss did not improve by at least 0.1 for 50 epochs. After initial evaluation, the best-performing model, selected based on per-OOI performance, was re-trained on the same data split and architecture using full-resolution images (as acquired during clinical imaging) to better preserve small structures (e.g., optic nerve, optic chiasm). This “Refined” model was generated via five-fold cross-validation with random sampling of the training and tuning images, producing five models whose voxel-wise predictions were averaged and softmax-normalized to yield the final ensemble output (Supplementary Material).

#### 2.2.4 SCARF 3: quantitative performance evaluation

Performance was evaluated using the hold-out testing set of 59 patient scans from RADCURE, estimated by averaging volumetric overlap indices, Dice similarity coefficient (DICE), and boundary distance metrics calculated using the resampled voxel sizing, 95th% Hausdorff Distance (95HD) [64]. The Refined model was evaluated using five quantitative performance metrics [64]: (1) Surface Distance - directed (SD); (2) Added path length (APL); (3) False Negative Volume (FNV); (4) False Negative Length (FNL); and (5) Jaccard index (IDX) (for equations see Supplementary Material).

#### 2.2.5 SCARF 4: clinical acceptability evaluation and surrogate benchmarking

Contours generated by the Refined model were evaluated for clinical acceptability through blinded expert review and correlation with quantitative metrics to develop a surrogate benchmarking model. We re-engineered **QUANNOTATE**, an open-source cloud-based QA tool [65], modifying it to assess contours by OOI category rather than by patient. Observers reviewed CT volumes slice-by-slice using an integrated web-based slider and could toggle between clinical windowing presets (bone, soft tissue, and lung).

Four expert observers, either medical fellows specializing in HNC malignancies or staff radiation oncologists with more than 10 years' HNC experience, rated contours using a 5-point Likert scale: 1 – very poor (unusable), 2 – poor (needs major edits), 3 – neutral (minor edits needed), 4 – good (within inter-physician variation), 5 – perfect (indistinguishable from expert-drawn) (Supplementary Material).

Observers were blinded to contour sources. For each of 19 OOIs, 20 cases were randomly selected from the 59-patient testing set (total: 380 contours per observer). Mean Acceptability Ratings (MAR) were computed by averaging scores across all four observers and 20 examples per OOI.

#### 2.2.6 SCARF 5: Generalizability assessment

Our Refined model was trained on the largest single institutional dataset, RADCURE. Generalizability of our model performance was tested using the remaining eight curated datasets from different institutions (Table 1).

Original dimensions of RADCURE scans were 512 × 512 (x-y plane), the relevant anatomy was found in more than half of the image, hence image cropping boosts inference efficiency. Otsu's thresholding [66] was used to create a ‘mask’ of the body, from which the patient's centre of mass was calculated and used to crop the image in the x-y plane with dimensions of 292 × 292. Image spacing was not standardized; original FOVs were used during inference to reflect routine clinical contouring without image manipulation or artifact introduction.

### 2.3 Use case

To demonstrate how the pre-trained models created using SCARF can be used for benchmark comparisons, we implemented and evaluated nnUNet [67]. The nnUNet model was trained using the 3D full resolution setting with 5-fold cross-validation for 1000 epochs, employing Dice-Cross Entropy Loss. After training, the performance of the model was compared to our previously best selected Refined method, with generalizability and clinical acceptability assessment.

### 2.4 Research reproducibility and reusability

To promote research reproducibility and reusability, our framework, computer code, and data table have been made publicly available under the Apache2.0 License at https://scarfai.ca/ via links to our public GitHub repository (https://github.com/bhklab/SCARF), Data Table and Google Colab notebooks. RADCURE is available on The Cancer Imaging Archive (TCIA) [49].

## 3 Results

### 3.1 SCARF 1: dataset curation

In RADCURE, 582 HNC patients had complete delineations for the 19 essential OOIs (Supplementary Fig. S2). We found extensive variability of ground truth information, where only a subset of the 19 OOIs of interest overlapped with any given dataset between RADCURE and the eight external datasets, spanning 701 patients (Supplementary Table S1). Three datasets, TCIA-HNSCC, SegRap2023, and HaN-Seg, had the most overlapping OOI categories with the RADCURE dataset (16 out of 19 OOIs successfully overlapped). Radiomics-HN1 had the least overlapping categories (5 out of 19 OOIs) (Supplementary Fig. S3).

### 3.2 SCARF 2 and 3: model training and performance evaluation

**Fig. 2:**
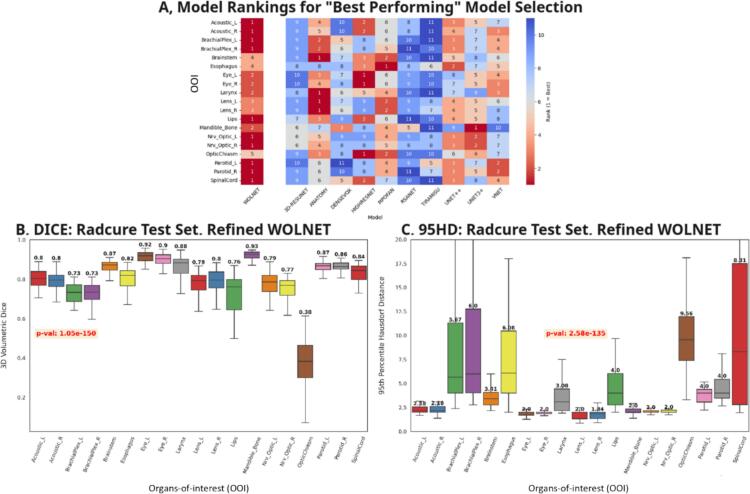
Selection and results of the best performing model: Refined WOLNET. A) a heatmap showing the associated ranking performance of a model for each OOI. WOLNET was the only model to rank in the top 5 for all OOIs, and top 3 for 16 of the 19 OOIs. B) DICE values for our Refined WOLNET model for each OOI category, and C) 95HD values for our Refined WOLNET model for each OOI category.

When analyzing the median performance metrics of all OOIs for each open-source model trained, the top three segmentation models, as determined by the weighted average of the highest median DICE and lowest median 95HD, were UNET variants. WOLNET was selected as the best performing model among the 12 evaluated models. WOLNET was the only model to rank in the top 5 for each OOI category, and top 3 for 16 of the 19 OOIs (Fig. 2 and Supplementary Fig. S4). ANATOMY and PIPOFAN were the second and third top-performing segmentation models. MedSAM2, a foundation model, ranked eighth of 12 models.

The Refined WOLNET model, the best performing open-source network, resulted in a final median test 95HD of (2.8 mm ± 14.8 mm) across all OOI(s) (Fig. 2). Lenses (2.2 mm ± 9.6 mm), eye (2.0 mm ± 1.1 mm) and optic nerves (2.0 mm ± 1.5 mm) had the lowest 95HD, whereas spinal cord (8.3 mm ± 18.5 mm), esophagus (6.1 mm ± 10.2 mm) and chiasm (9.6 mm ± 3.5 mm) had the highest recorded 95HD. Mandible and parotid gland (R) had higher variance (*p* < 0.05) in 95HD compared to other OOIs.

### 3.3 SCARF 4: clinical acceptability assessment

**Fig. 3:**
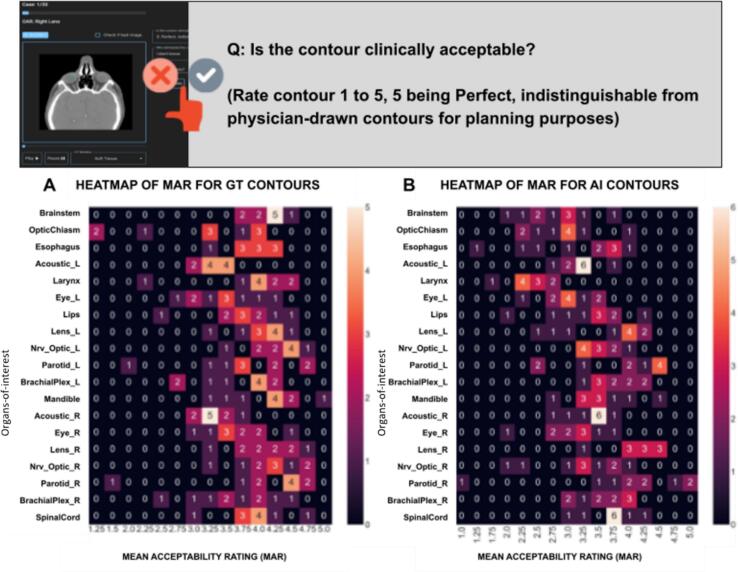
Results of refined WOLNET clinical evaluation recording using quannotate QA tool. Results of the acceptability test by representing mean Likert rating counts for each OOI in a heat-map for A) ground-truth contours (GT) and B) AI-generated contours (AI). The higher the value of a box the more contours of that given OOI (row) had any given Likert rating (column) and the lighter the corresponding box appears.

Mean acceptability rating for the ground-truth contours showed a global average of 3.8 ± 0.9 compared with 3.4 ± 1.0 for AI-generated contours. Additionally, when comparing MAR for all OOIs, 78% of ground-truth contours were considered acceptable (MAR > 3.5) (Fig. 3A) compared with 52% for AI-generated contours (Fig. 3B).

In individual OOI categories, ground-truth contours were considered more acceptable than AI-generated contours for 15 out of the 19 OOIs assessed. The least clinically acceptable ground-truth OOI was the chiasm with a MAR of 3.0 ± 1.3, while the most acceptable was the mandible with a MAR of 4.2 ± 0.9. For AI-generated contours the least and most acceptable contours were larynx (MAR = 2.4 ± 0.9) and the lenses (MAR of 3.9 ± 0.9), respectively.

Experts rated 16 out of 19 AI-generated OOIs as acceptable for planning with minor edits (3 < MAR < 3.5) (Table 2). Only three OOI categories (brainstem, larynx, and the right optic nerve) were shown to be rated more clinically acceptable than their paired deep learning contour with sufficient post-hoc power (PHP > 80%). Ten OOI categories had no significant differences in MAR (PHP < 20%). The MAR between the remaining six OOI categories (20% < PHP < 80%) may be significantly different if more samples are analysed for each group (Table 2).

**Table 2:** MC v. DLC for each refined WOLNET OOI category. Pn80 is defined as the minimum power required (number of paired samples) to have a significantly different rating between ground-truth and AI contours. For example, for our analysis of the sample size, when assessing whether certain OOI categories passed the mean acceptability cutoff of 3.5, 15 manually delineated OOIs on average were considered clinically acceptable, requiring no edits for planning purposes, compared with 9 OOIs generated by deep learning. When analyzing categories of OOIs requiring minor edits for their contours to be accepted into radiation therapy plans (3.0 < MAR < 3.5), 7 deep learning generated OOIs compared with 4 manually contoured OOIs met this criteria.

ROI	MAR (GT)	MAR (AI)	Pn80	PHP %	ROI	MAR (GT)	MAR (AI)	Pn80	PHP %
Mandible	**4.15** **±** **0.89**	3.53 ± 1.13	32	27.5					
Eye_L	**3.45** **±** **0.71**	3.00 ± 0.78	39	27.1	Lens_R	4.10 ± 0.78	**4.15** **±** **0.70**	3820	3.5
Eye_R	**3.68** **±** **0.62**	3.05 ± 0.68	15	58.1	Acoustic_L	**3.30** **±** **0.91**	3.20 ± 0.97	1300	4.3
Brainstem	**4.13** **±** **0.69**	2.8 ± 0.91	**4**	**95.8**	Acoustic_R	3.30 ± 0.72	**3.38** **±** **0.74**	1272	4.3
Larynx	**3.95** **±** **0.85**	2.38 ± 0.90	**5**	**98**	BrachialPlex_L	3.68 ± 0.76	**3.78** **±** **0.58**	907	5.2
SpinalCord	**3.93** **±** **0.76**	3.70 ± 0.56	171	11.7	BrachialPlex_R	**3.63** **±** **0.84**	3.58 ± 0.81	4431	3.4
Parotid_L	3.80 ± 0.99	**3.85** **±** **1.02**	6154	3.2	Lips	**3.75** **±** **0.71**	3.33 ± 0.80	45	23.6
Parotid_R	**4.08** **±** **1.02**	3.95 ± 1.23	966	4.4	Nrv_Optic_L	**4.28** **±** **0.68**	3.50 ± 0.78	12	66.47
Esophagus	**3.93** **±** **0.80**	3.13 ± 0.97	16	52.1	Nrv_Optic_R	**4.18** **±** **0.68**	3.20 ± 0.79	**8**	**84.5**
Lens_L	**4.05** **±** **0.68**	3.65 ± 0.92	45	19.6	OpticChiasm	**3.03** **±** **1.27**	2.90 ± 1.06	1498	4.3

### 3.4 SCARF 5: generalizability assessment

**Fig. 4:**
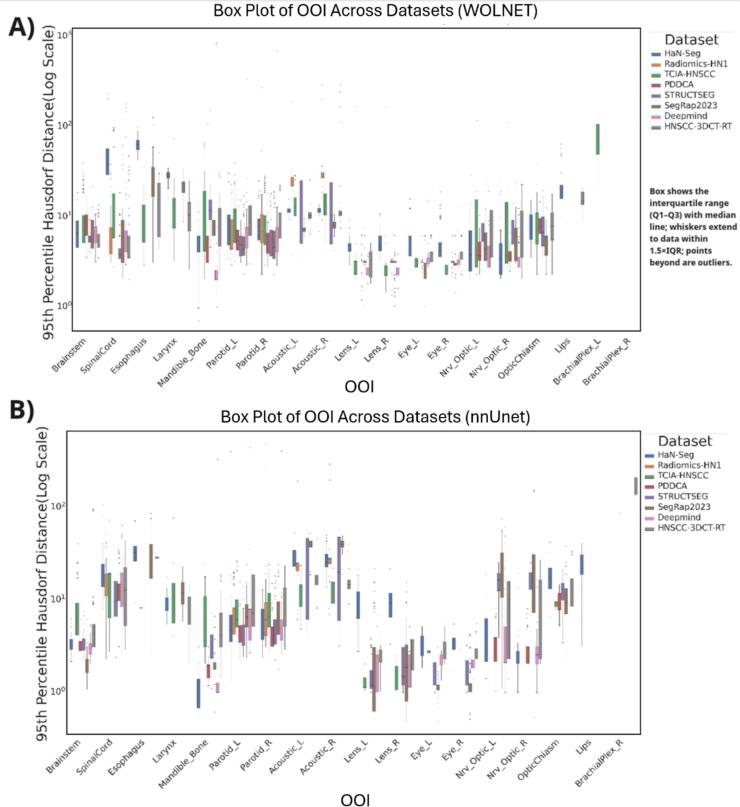
Generalizability Assessment of Refined WOLNET and nnUNet. Box plots to show variation of 95HD of the Refined WOLNET and nnUNet across eight external datasets. A) 95HD for Refined WOLNET, and B) 95HD for nnUNet.

Comparison of the performance (DICE and 95HD) of the Refined model to nnUNet across all external datasets are found in Fig. 4 and Supplementary Fig. S5. Supplementary Tables S3 and S4 show the OOI specific DICE and 95HD for each external dataset.

The top performing OOI category for the Radiomics-HN1 dataset was the mandible with a median 95HD of 4.0 mm ± 2.0 mm; the poorest performing OOI (with more than one ground-truth mask present in the dataset) was the acoustics, with median 95HD of 7.0 mm ± 10.4 mm. The HNSCC-3DCT-RT (H3DR) and StructSeg19 (SS19) datasets had 14 out of the 19 RADCURE OOIs delineated. The top performing OOI categories in H3DR were the eye (L/R), with median 95HD scores of 2.8 mm ± 0.9 mm and 2.5 mm ± 0.6 mm respectively; the poorest performing OOI in H3DR was the chiasm achieving a median 95HD of 5.1 mm ± 3.5 mm. The top performing OOI categories in SS19 were the parotid glands (L/R) both achieving median 95HD scores of 3.0 mm ± 1.7 mm and 3.0 mm ± 3.0 mm respectively. The poorest performing OOI in SS19 was also the chiasm with a median 95HD of 5.1 mm ± 3.5 mm.

In generalizability assessment, model performance was lower than internal hold-out validation, potentially due to various institutional characteristics associated with patient demographics, image collection, and/or clinical contouring guidelines, making it challenging to determine the root cause(s) of the lowered performance.

### 3.5 Use case – nnUNet benchmarking

The nnUNet yielded a 95HD across all OOIs of 2.0 mm ± 9.7 mm. This was a substantial improvement over the Refined WOLNET which had a 95HD of 2.8 mm ± 14.8 mm. The best performing OOI was the mandible, with a 95HD of 1.4 mm ± 13.5 mm. The poorest performing OOI was the optic chiasm, with a 95HD of 2.2 mm ± 1.8 mm. All OOIs segmented by the nnUNet model yielded a lower 95HD compared to the Refined WOLNET.

Generalizability assessment of nnUNet demonstrated similar performances, with higher performance on the external datasets. Some outliers included the Acoustic structures in the SegRap dataset, which exhibited a significant performance drop when comparing the Refined WOLNET to nnUNet (Supplementary Fig. S5). The best performing OOI across all eight datasets was the mandible in the HaN-Seg dataset with a 95HD of 1.1 mm ± 0.6 mm. The worst performing OOI across all eight datasets was the Acoustic(L/R) in the SegRap2023 dataset with a median 95HD of 39.0 mm ± 5.0 mm.

## 4 Discussion

We introduced SCARF, a five-step benchmarking framework for evaluating open-source AI models in the auto-segmentation of OOIs in radiotherapy for HNC. SCARF identified WOLNET as the best performing model among 12 open-source models utilizing 95HD and DICE. AI-generated contours were clinically acceptable with only minor revisions for 16/19 OOIs.

SCARF is modular, flexible, and scalable, allowing adaptation to varying research needs. The Clinical Acceptability and Generalizability Assessment steps may be performed in either order depending on dataset availability and reviewer readiness. Alternative data curation pipelines, such as the scalable infrastructure proposed by Tryggestad et al. [68], can be integrated. Furthermore, SCARF's clinical acceptability component can be extended with dose/volume metric evaluation, as in the work of Ye et al. [69].

A key innovation in SCARF is its emphasis on clinical acceptability. While many benchmarking frameworks focus solely on quantitative performance metrics, SCARF incorporates expert review to evaluate contours' fitness for clinical use. To mitigate the high cost and resource demand of clinical assessment, SCARF includes a clinical acceptability surrogate benchmark, allowing users to screen models prior to investing in full expert review.

Though dose/volume metric analysis is a standard component of model evaluation, it was intentionally excluded from SCARF in its current form. Clinical consultation suggested prioritizing anatomical accuracy over surrogate measures of dose/volume metric impact, especially in IMRT/VMAT planning where dose to OOIs is strongly influenced by anatomical definition [70]. Nevertheless, we recommend that users perform dose/volume metric evaluation before clinical deployment, and we plan to incorporate this in future SCARF updates.

There are several limitations to our current implementation. Only cases with full annotation of 19 OOIs were included, reducing the number of usable training and testing samples. Future work will address this by leveraging newer methods for handling incomplete data, enabling more comprehensive use of the RADCURE dataset.

A consistent custom loss function was applied across models to standardize training; however, this required adapting some architectures from 2D to 3D and may not reflect each model's originally intended optimization. Future applications of SCARF will allow models to use their native loss functions for a more faithful comparison.

We expect inter-clinician variability in clinical acceptability rating as it relies on a subjective, 5-point Likert scale, and perceptions of contour acceptability will vary among clinicians. Despite inter-clinician variability, subjective ratings are correlated with clinical outcomes [24], [71]. However, clinical acceptability testing was performed by only four radiation oncologists, limiting statistical power. Significant differences in acceptability were found in only three of nineteen OOI categories. Broader clinician participation is needed for more robust conclusions.

We note the volume dependence of DICE [24], [72], a commonly used metric in segmentation evaluation. Smaller structures tend to receive lower DICE scores even with small deviations, complicating cross-OOI comparisons. In selecting a model for refinement, we relied on median performance, but presented per-OOI results to highlight variation in performance across structures of different sizes. We encourage SCARF users to consider global and local metrics when comparing models and tailor metric selection to the specific comparison task.

SCARF provides the first structured, open-source protocol for end-to-end evaluation. It is designed to produce standardized, reproducible benchmarking results across diverse AI models and datasets to validate models prior to implementation in a clinical setting.

## CRediT authorship contribution statement

**Joseph Marsilla:** Writing – original draft, Validation, Software, Resources, Methodology, Investigation, Formal analysis, Data curation, Conceptualization. **Mattea L. Welch:** Writing – review & editing, Writing – original draft, Visualization, Validation, Supervision, Software, Resources, Project administration, Methodology, Investigation, Formal analysis, Data curation, Conceptualization. **Joshua Siraj:** Writing – review & editing, Writing – original draft, Validation, Software, Resources, Methodology, Investigation, Formal analysis, Data curation. **Clare McElcheran:** Writing – review & editing, Visualization, Supervision, Project administration, Investigation. **Jun Won Kim:** Visualization. **Denis Tkachuck:** Software. **Sejin Kim:** Writing – review & editing, Software, Methodology, Data curation, Conceptualization. **John Cho:** Software. **Ezra Hahn:** Resources, Data curation. **Ali Hosni:** Supervision, Resources, Funding acquisition. **J.C. Jacinto:** Resources, Data curation. **Michal Kazmierski:** Resources, Data curation. **Katrina Rey-McIntyre:** Resources, Formal analysis, Data curation. **Shao Hui Huang:** Resources, Data curation. **Tirth Patel:** Software, Data curation. **Tony Tadic:** Supervision, Software, Resources, Data curation. **Scott Bratman:** Data curation, Conceptualization. **Andrew Hope:** Writing – review & editing, Supervision, Resources, Methodology, Funding acquisition, Data curation, Conceptualization. **Benjamin Haibe-Kains:** Writing – review & editing, Supervision, Resources, Project administration, Methodology, Funding acquisition, Data curation, Conceptualization.

## Funding statement

None to report.

## Declaration of competing interest

The authors declare that they have no known competing financial interests or personal relationships that could have appeared to influence the work reported in this paper.
