# Supplementary material for: Auto-segmentation of organs-of-interest clinical acceptability & reproducibility framework in head and neck cancer
Source: Phys Imaging Radiat Oncol. 2026 Jun 6;39:101015. doi: 10.1016/j.phro.2026.101015 (PMC13312076; doi:10.1016/j.phro.2026.101015)
Supplement: Supplementary file 1 — Supplementary material [file mmc1.pdf]

Supplementary Material

Model Training

The RADAM optimizer was used to minimise the loss during training [1,2]. The initial learning rate of the optimizer was set to .001. The tuning loss was monitored and the learning rate decreased by a factor of 0.50 if there was no significant change in tuning loss after 12 epochs. The voxel spacing of each patient scan was standardised to a lower resolution of 1 mm x 1 mm x 3 mm using SimpleITK [3].

Translation, mirroring, zooming, and rotation were applied in-plane (x-y plane only) at random during training. We used random translations between -32 and 32 pixels in each plane; uniform scaling factors between 0.9 and 1.1; and mirrored images with a probability of 0.50. Patient scans were cropped to a size of (192 x 192 x 64) pixels for use during training. The ground truth mask was first used to identify the coordinates of the patient's centre of mass on which cropping was based. All CT volumes used in analysis were clipped to a HU range of -200 to 300 before augmentations were applied. This window was chosen to visualize fat (approx. -100 HU), soft tissue (approx. 20-80 HU) and to avoid bone dominance (>300 HU) [4]. Z-score normalisation was applied after all augmentations were completed.

Early stopping was consistent across all models and was triggered if tuning loss did not improve by at least 0.1 for 50 epochs. Total trainable parameters for each model varied as follows: 9181390 for WOLNET; 3049070 for UNET++; 8539813 for PIPOFAN; 1994931 for ANATOMYNET; 30377088 for VNET; 115552 for HIGHRESNET; 35317332 for 3D-RESUNET; 26119907 for RSANET; 1784305 for DENSEVOXNET; 79982305 for UNET3+; and 989280 for TIRAMISU. Evaluation was performed at the last checkpoint.

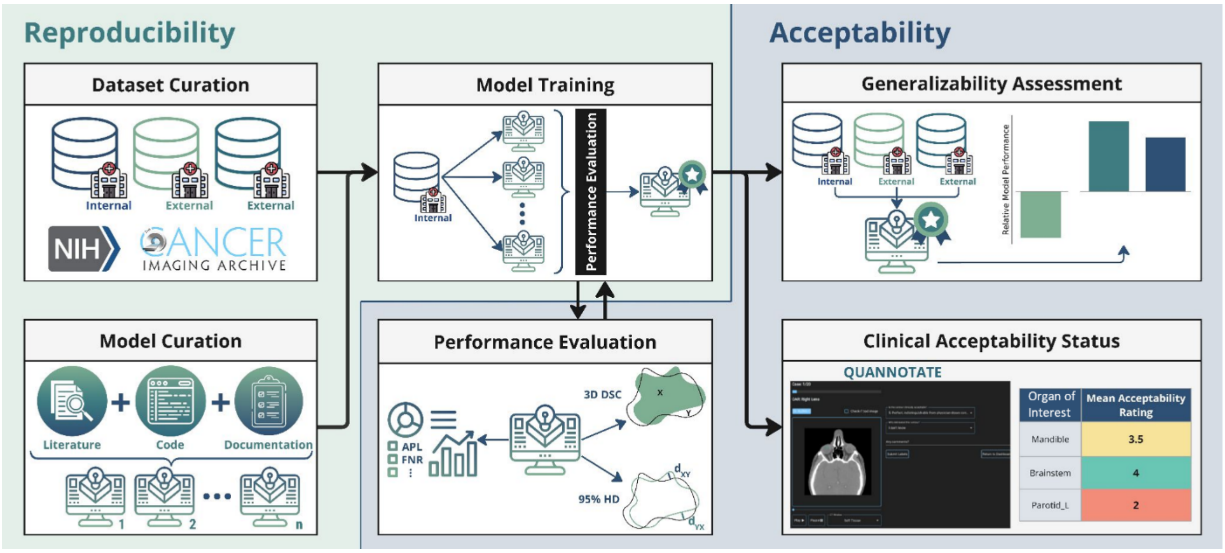

Figure S1: SCARF Overview: auto-Segmentation Clinical Acceptability & Reproducibility Framework. Reproducibility: The first half of the framework emphasizes reproducible

development of code and software used during dataset curation and model training stages. Acceptability: In the second portion of the framework, emphasis is placed on development of an acceptability standard that uses quantitative performance evaluation, clinical acceptability assessment, and generalizability assessment.

Model Fine-tuning

The best model was selected for a 2nd re-training phase on full-resolution patient scans for an additional three days on 4 NVIDIA Tesla V100 GPUs and tested using MONAI based sliding-window inference [5]. The initial learning rate was 4e-4; tuning loss was monitored and the learning rate was decreased by a factor of 0.96 if there was no significant change in loss for 1 epoch. All CT volumes were clipped to a HU range of -500 to 1000. Cropping regimen was modified to 192 x 192 x 128 pixels. Images were not resampled for re-training. We used random translations between -64 and 64 pixels, the other randomised augmentations and Z-score normalisation scheme were unchanged. The initial convolutional map was set to 48 feature maps.

Model Evaluation

All open-source models explored in this study were evaluated using Sorensen-Dice coefficient Calculation (DICE), and Hausdorff distance of the 95th percentile (HD95). DICE looks at the volumetric overlap of the ground-truth and inferred contours. A DICE of 0 represents no overlap, with 1 representing perfect overlap. It is calculated using custom code that implements the following equation where X represents the ground-truth contour voxels and Y represents the inferred contour voxels $DICE = \frac{2|X \cap Y|}{|X| + |Y|}$. HD95 calculates the 95th percentile of the distances (in millimeters) between the boundaries of Volume X and Y, this minimises the impact a small subset of outliers could have on distance calculations. Perfect overlap of the contours is a value of 0. It is calculated using the MONAI (https://monai.io/) implementation of the following equation $d_H(X,Y) = max\{d_{XY}, d_{YX}\} = max\{max_{x \epsilon X} min_{y \epsilon Y} d(x,y), max_{y \epsilon Y} min_{x \epsilon X} d(x,y)\}$.

For the Refined best performing model we also calculated five additional performance metrics [6]: (1) Jaccard Index, also known as the Intersection-Over-Union, is implemented using custom code. It is calculated as $J(X,Y) = \frac{|X \cap Y|}{|X \cup Y|}$; (2) Surface Distance (directed, by default), $\frac{2|X \cap Y|}{|X| + |Y|}$ where X and Y are surfaces, is calculated using MONAI (https://monai.io/); (3) Added Path Length $X - (X \cap Y)$, where X is the ground-truth surface and Y is the auto-segmented surface (measured in voxels); (4) False Negative Path Length, $X - (X \cap Y)$, where X is the ground-truth surface and Y is the auto-segmented volume (measured in voxels); and (5) False Negative Volume, $X - (X \cap Y)$, where X is the ground truth volume and Y is the auto-segmented volume (measured in voxels). Both Added Path Length and the False Negative Volume are calculated using open-source code from the Auto-Segmentation Spatial Similarity Metrics code base (https://github.com/kkiser1/Autosegmentation-Spatial-Similarity-Metrics/). A Kruskal-Wallis H-test was used to determine statistical significance between model performance for each OOI. Results are reported with a p-value.

For Mean Acceptability Ratings, observers assessed the clinical acceptability of a contour by rating it on a 5-point Likert clinical acceptability scale where ratings were described as: 1-Very poor, large areas need minor or major edits, is unusable for planning purposes; 2-Poor, needs significant edits to be used for planning purposes; 3-Neutral, needs minor edits to be used for planning purposes; 4-Good, within acceptable inter physician variation for planning purposes; and 5-Perfect, indistinguishable from physician drawn contours for planning purposes.

Contents of Radcure Cohort Used In Analysis

The study cohort consisted of 378 oropharyngeal, 123 nasopharyngeal, 10 hypopharyngeal, 10 oral cavity, and 7 laryngeal cancer patients, as well as 55 patients of unknown or other primary-site cancers. The 19 OOIs included acoustics (L/R), brachial plexuses (L/R), brainstem, chiasm, oesophagus, eyeballs (L/R), larynx, lenses (L/R), lips, mandible, optic nerves (L/R), parotid glands (L/R), and spinal cord. Excluded OOIs included Lacrimal Gland (L/R), Spinal Canal, Brain, Submandibular Gland (L/R), Oral Cavity, Trachea, Pituitary Gland, Constrict Muscles (S/M/I), Cricoid, and Anterior Chamber.

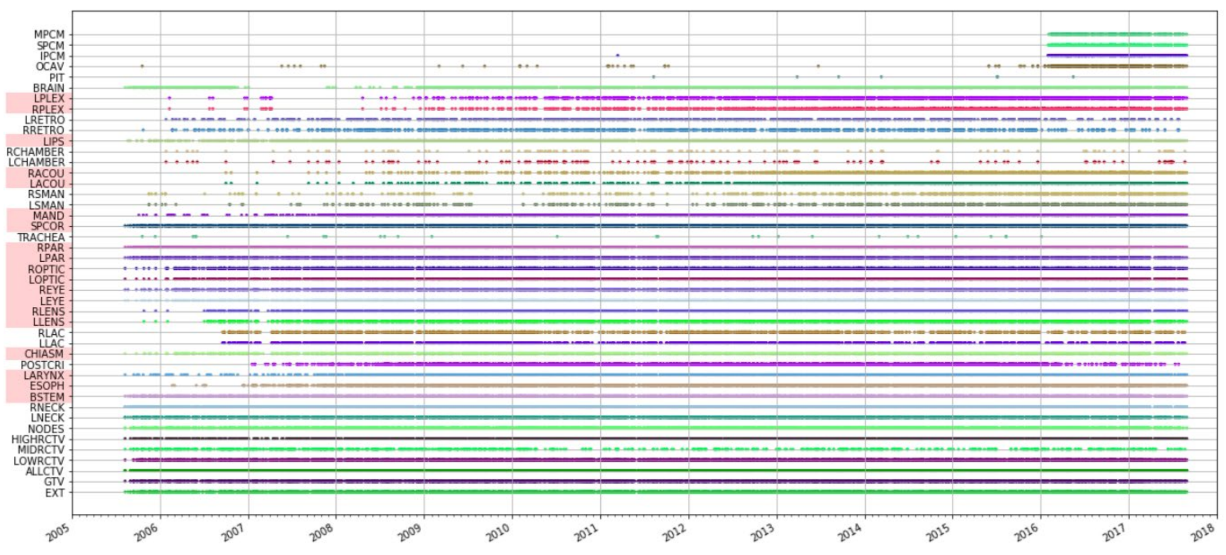

Figure S2: Availability of OOI segmentation in our cohort of 3,251 HNC patients included in our RADCURE dataset. A subset of 582 patients have 19 OOIs fully segmented (highlighted in red), this set was used to train and test our compendium of 12 open-source auto-segmentation models.

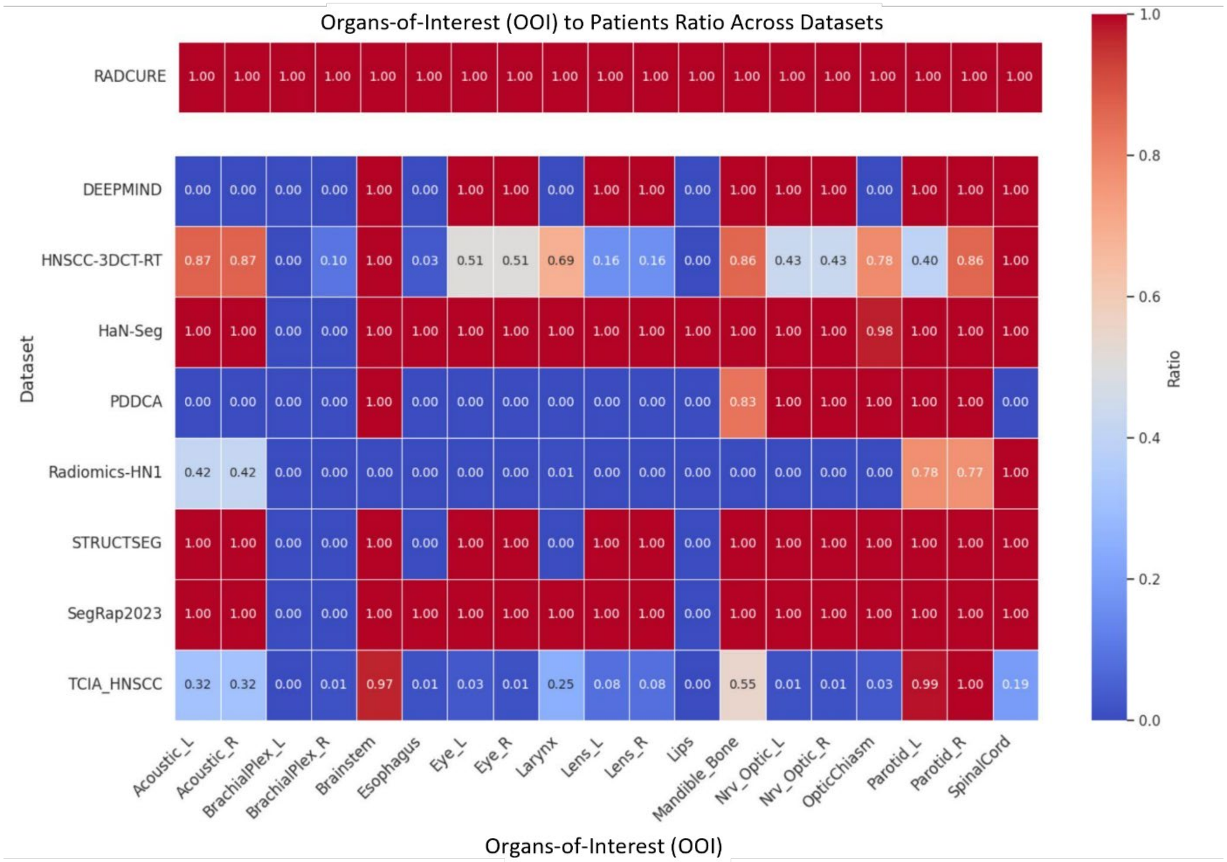

Figure S3: External dataset OOI availability. 19 OOIs were selected for training our model out of the available 34 OOIs in RADCURE. This allowed for a "complete" dataset to be used where each patient had all OOIs of interest, and no missing data. For the external validation datasets the availability of the 19 OOIs is displayed as a ratio where 1 means that the OOI is available for all patients in that dataset, 0.5 means that the OOI is available for 50 % of the patients in that dataset, and 0 means that OOI is unavailable for any patients in that dataset.

Table S1: Distribution of Ground Truth OOI labels (masks) in external datasets

OOI	RADCURE (n=59)	HNSCC-3DCT-RT (n=87)	Deepmind - Onc (n=35)	Deepmind- Rad (n=35)	PDDCA (n=48)	Radiomics-HN1 (n=129)	STRUCTSEG(n=50)	TCIA_HNSCC (n=190)	SegRap2023 (n=120)	HaN-Seg(n=42)
BSTEM	59	82	35	35	48	0	50	181	120	42
CHIASM	59	71	0	0	48	0	50	15	120	41
ESOPH	59	12	0	0	0	0	0	20	120	42
LACOU	59	78	0	0	0	54	50	58	120	42
LARYNX	59	56	0	0	0	0	0	46	120	42
LEYE	59	56	35	35	0	0	50	7	120	42
LIPS	59	8	0	0	0	0	0	0	0	42
LLENS	59	13	35	35	0	0	50	22	120	42
LOPTIC	59	37	35	35	48	0	50	10	120	
LPAR	59	81	35	35	48	101	50	188	120	42
LPLEX	59	0	0	0	0	0	0		0	
MAND	59	70	35	35	48	0	50	107	120	42
RACOU	59	79	0	0	0	54	50	61	120	42
REYE	59	59	35	35	0	0	50	61	120	42
RLENS	59	0	35	35	0	0	50	22	120	42
ROPTIC	59	38	35	35	48	0	50	10	120	42
RPAR	59	81	35	35	48	100	50	188	120	42
RPLEX	59	0	0	0	0	0	0		0	
SPCOR	59	86	35	35	0	129	50	43	120	42

Model Curation

Many networks cannot be reproduced easily because of various computational or organisational hurdles. In addition, many publications fail to compare findings to validated baseline models. Complex network modifications may only prove useful if they are compared to a simple baseline architecture. To address the lack of open-source resources to validate findings of previously published studies, we performed a large comparative analysis of open-source deep neural networks applied to image segmentation in a medical context. We looked for additional studies published on a medical image segmentation task to collect open-source networks that could be

trained on our OOI segmentation task. A total of 2D networks that could not be converted to 3D convolutional scheme without modifying the architectural integrity, close similarity or overlap between other architectures, code released with the study was unmaintained and/or could not be integrated into PytorchLightning.

Table S2: Architectures selected for integration to SCARF code-base for easy re-implementation, training & validation.

Name	Description
**3D-UNET (WOLNET)**	One popular pytorch implementations of the standard 3D UNET paper [7] were taken and used as the baseline architecture. Named WOLNET after its author. [8]
**3D-RESUNET**	We integrated a third party implementation [8] of a residual symmetric 3D UNET proposed by [9]. They introduced a residual skip connection to each valid convolutional module present in their network. To minimise information loss, this network does not down sample feature maps along the z dimension. To minimise the effects of anisotropy 2D convolutions are used in the modules at the lowest part of the network, which contain fine scale feature maps. Each regular residual module will apply in total 7x7x5 of nonlinear convolutions to the input. (To embed 2D features 3x3x1 convolutions are applied followed by subsequent 3x3x3 convolutions.).
**HIGHRESNET**	Li, W et al [10] choose to integrate dilated convolutions and residual connections in their proposed 20 layer residual network. These residually connected dilated convolutions allowed for multi-scale feature preservation as training progressed [10]. A third party implementation of HighResNet3D was used for our analysis [11]**.**
**PIPOFAN**	We modified the original 2D Pyramid Input Pyramid Output Network proposed by Fang, et al [12] to accept 3D volumes. This 3D pyramid abstraction network (PIPOFAN) processes the volumetric input by applying 3D Equal Depth Convolutions (EDC), after passing through the network the pyramid outputs are fused together which has been shown to improve subsequent organ segmentations. This network was originally used to segment multiple thoracic OOIs on individual slices of a CT scan [12].
**UNET3+**	We introduced a 3D version of a 2D UNET3+ proposed by Huang, H et al [13] which was created as a modification of the UNET++ that incorporated full-scale skip-connections into the UNET++ network. This architecture was engineered to produce full-scale aggregated feature maps that allows deep supervision components to learn more comprehensive hierarchical feature maps with the hopes of producing more accurate contours [13]**.**
**UNET++**	We integrated a 3D version of the 2D Nested Unet Architecture (UNET++) proposed by Zhou et al [14] using their code as the base network for the architecture used in the study. The authors redesigned sip connection pathways of the original UNET architecture with the intent to reduce the semantic gaps between the encoding and decoding feature maps. This is the first paper to propose and integrate deep supervision into their network where the outputs of each individual segmentation branch are averaged before the final softmax layer of the network [14].
**ANATOMY**	AnatomyNet was the only segmentation model used in this analysis that was previously published on a HNC OOI segmentation task. This UNET variant incorporates squeeze and excitation residual building blocks in the downsampling/upsampling layers of the network. Code was refactored and updated to suit the newest versions of pytorch [15].

**DENSEVOX**	A pytorch based third party implementation of DenseVoxNet 3D first proposed by Yu, L et al [16], for cardiac segmentation was integrated into our study. This network consists of two DenseBlocks in the downsampling part of the network which are densely connected. In total there are 24 transformation layers before upsampling. A long skip connection was used to stabilise the training process by connecting the transition layer to the output layer [11,16].
**TIRAMISU**	We adapted a third party 2D implementation of a fully convolutional Densenet originally presented by Jegou et al, [17] (named 100-layer tiramisu). This paper was the first to apply the DenseNet to the problem of 2D semantic segmentation. Densenets are constructed by concatenating each output of a subsequent densely connected convolutional block to the next block, therefore linearly augmenting the number of feature maps after each 'down transition'. This does not occur in the upsampling part of the network. The feature maps from the downsampling path are then concatenated with those of the upsampling path to produce a predicted segmentation mask at the resolution of the original input [17].
**RSANET**	RSANet is a 3D recurrent slice-wise attention network proposed by Zhang et al [18] could be directly integrated into our network. Originally constructed for Multiple Sclerosis lesion segmentation this network utilises slice wise attention blocks to help capture long-range inter-slice dependencies along any direction of a 3D medical image. These blocks allow for the recurrent aggregation of information along multiple directions therefore providing a mechanism to help capture global contextual information, which can be used to produce more accurate segmentations [18].
**VNET**	An updated third party implementation of 3D VNET architecture proposed by Millerari et al [19] was used in this study. The VNET architecture is a fully convolutional network based on the original UNET. The authors choose to replace 3x3x3 convolutions present in UNET by 1 strided 5x5x5 convolutions. Additionally, in place of max-pooling, 2x2x2 convolutions with stride of 2 were used during down sampling. Finally, PReLU nonlinearities were chosen to replace original ReLUs throughout the network [19].
**MEDSAM2**	An updated 3D adaptation of the Segment Anything Model 2 [20] that was fine-tuned on an extensive medical dataset containing over 455,000 3D image–mask pairs and tens of thousands of video [21]. This promptable segmentation foundation model was developed to generalise across a wide range of organs, lesions, and imaging modalities.

Model Performance Additional Metrics

The training scheme of the best performing open source network, WOLNET, was further refined resulting in a final median test DICE of (0.82 ± 0.15). Mandible (0.93 ± 0.03), Eyes (0.92 ± 0.03), and Brainstem (0.87 ± 0.04) received the highest median DICE, while Chiasm (0.38 ± 0.11), Lips (0.76 ± 0.21), and Brachial plexus (0.73 ± 0.14) received the lowest median DICE respectively. Lips and the Acoustics (L/R) had higher variance ($p<0.05$) in DICE compared to other OOIs.

Generalizability Assessment (Refined WOLNET)

To assess the generalizability of the Refined WOLNET model, we used 8 external datasets generated at different institutions. The top performing OOI category for the Radiomics-HN1 dataset was the mandible with a median DICE of 0.85 ± 0.05; the poorest performing OOI (with more than one Ground-Truth mask present in the dataset) was the Acoustics, with median DICE of 0.32 ± 0.17. The top performing OOI categories in H3DR were the eyeballs (L/R), both with median DICE scores of 0.83 ± 0.04 and 0.83 ± 0.05; the poorest performing OOI in H3DR was

the chiasm achieving a median DICE of 0.27 ± 0.21. The top performing OOI categories in SS19 were the parotids (L/R) both achieving median DICE scores of 0.86 ± 0.04. The poorest performing OOI in SS19 was also the Chiasm with a median DICE of 0.33 ± 0.17.

nnUNet Performance

The nnUNet yielded a median DICE and HD95 across all OOIs of 0.87 ± 0.12 and 2.0 mm ± 9.7 mm, respectively. This is a substantial improvement over the Refined WOLNET which had a DICE and HD95 of 0.82 ± 0.15 and 2.8 mm ± 14.8 mm, respectively. The best performing OOI was the Mandible, with a DICE and HD95 of 0.96 ± 0.03 and 1.4 mm ± 13.5 mm, respectively. The poorest performing OOI was the Optic Chiasm, with a DICE and HD95 of 0.76 ± 0.18 and 2.2 mm ± 1.8 mm, respectively. All OOIs segmented by the nnUNet model yielded a higher DICE and lower HD95 compared to the Refined WOLNET.

Generalizability assessment of nnUNet showcased similar performances, with higher performance on the external datasets. Some outliers included the Acoustic structures in the SegRap dataset, which exhibited a significant performance drop when comparing the Refined WOLNET to nnUNet (Supplementary Figure S5). The best performing OOI across all eight datasets was the Mandible in the HaN-Seg dataset with a median DICE and HD95 of 0.95 ± 0.02 and 1.1 mm ± 0.6 mm, respectively. The worst performing OOI across all eight datasets was the Acoustic(L/R) in the SegRap2023 dataset with a median DICE and HD95 of 0.18 ± 0.03 and 39.0 mm ± 5.0 mm, respectively.

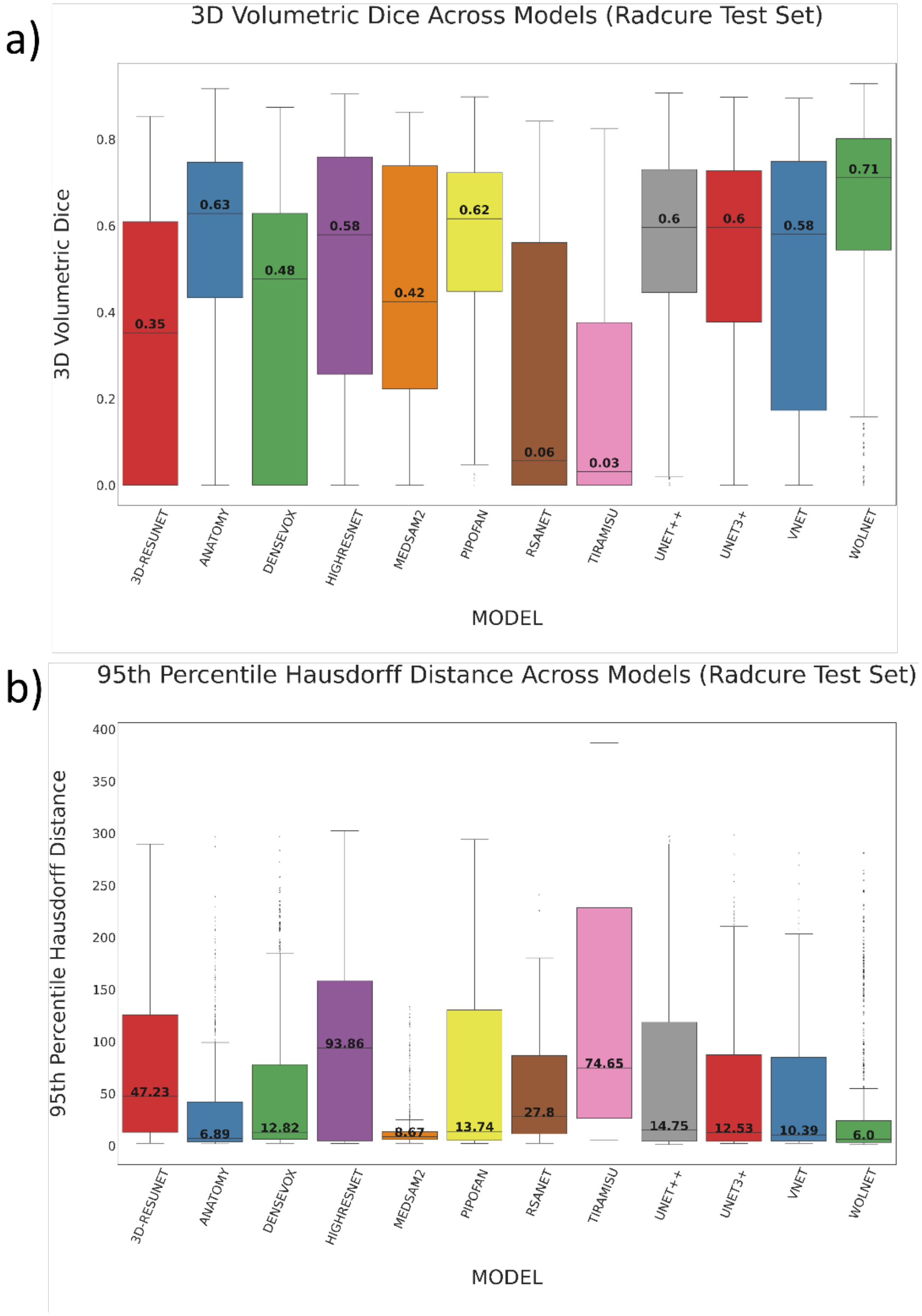

Figure S4: Performance Evaluation of Model Training. Eleven open-source networks were selected based on availability, complexity and adaptability. Each model was trained to auto-segment 19 OOIs using 523 patient scans, and evaluated using a hold out dataset of 59 patient scans. Barplots show performance metrics for each of the eleven models using: a) DICE, and b) 95HD. WOLNET had the best performance of the evaluated models and was selected for retraining and use in clinical acceptability testing.

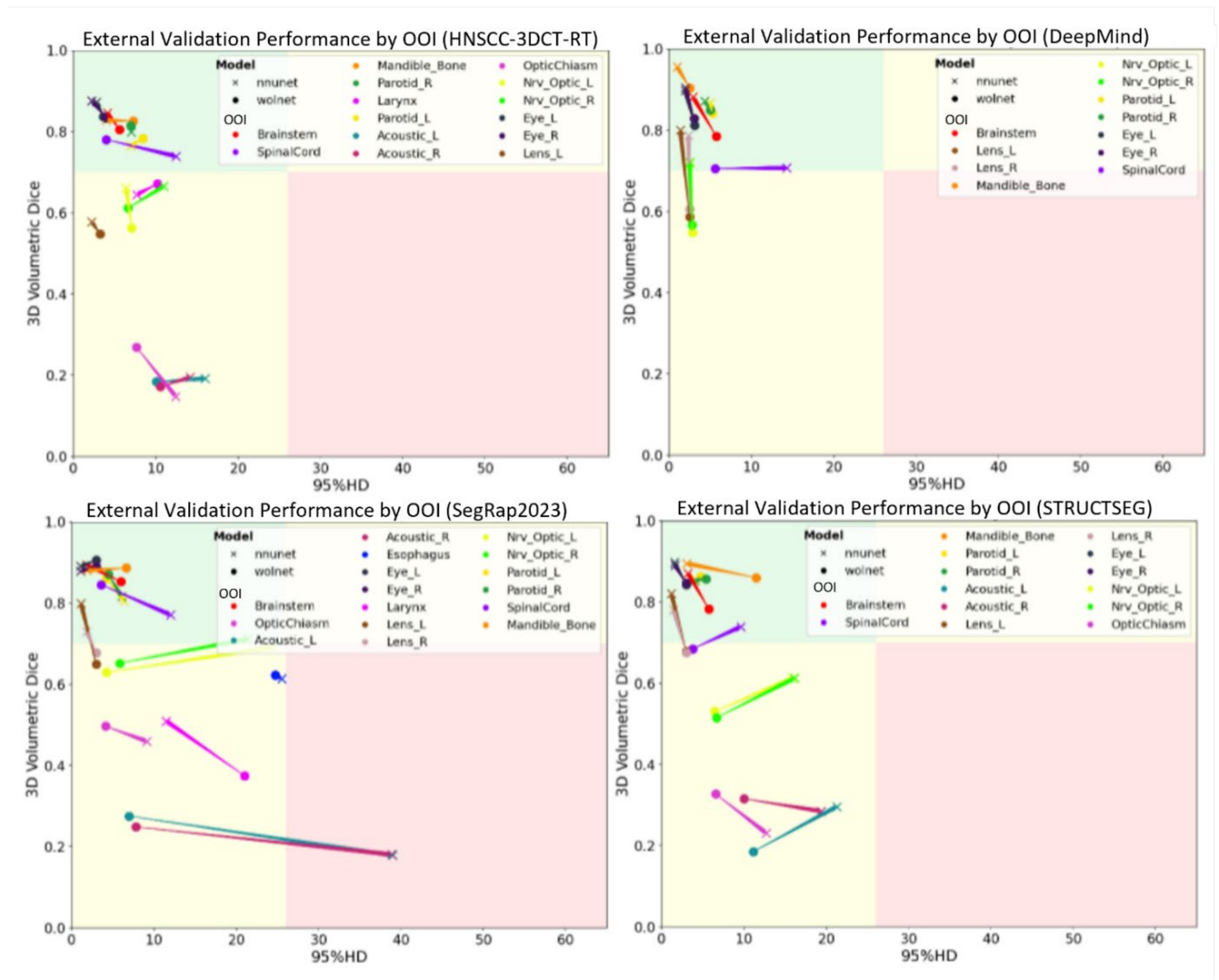
External Validation Performance by OOI (HNSCC-3DCT-RT)
Model
nnunet
wolnet
OOI
Brainstem
SpinalCord
Mandible_Bone
Parotid_R
Larynx
Parotid_L
Acoustic_L
Acoustic_R
OpticChiasm
Nrv_Optic_L
Nrv_Optic_R
Eye_L
Eye_R
Lens_L
3D Volumetric Dice
95%HD
External Validation Performance by OOI (DeepMind)
Model
nnunet
wolnet
OOI
Brainstem
Lens_L
Lens_R
Mandible_Bone
Nrv_Optic_L
Nrv_Optic_R
Parotid_L
Parotid_R
Eye_L
Eye_R
SpinalCord
3D Volumetric Dice
95%HD
External Validation Performance by OOI (SegRap2023)
Model
nnunet
wolnet
OOI
Brainstem
OpticChiasm
Acoustic_L
Acoustic_R
Esophagus
Eye_L
Eye_R
Larynx
Lens_L
Lens_R
Nrv_Optic_L
Nrv_Optic_R
Parotid_L
Parotid_R
SpinalCord
Mandible_Bone
3D Volumetric Dice
95%HD
External Validation Performance by OOI (STRUCTSEG)
Model
nnunet
wolnet
OOI
Brainstem
SpinalCord
Mandible_Bone
Parotid_L
Parotid_R
Acoustic_L
Acoustic_R
Lens_L
Lens_R
Eye_L
Eye_R
Nrv_Optic_L
Nrv_Optic_R
OpticChiasm
3D Volumetric Dice
95%HD

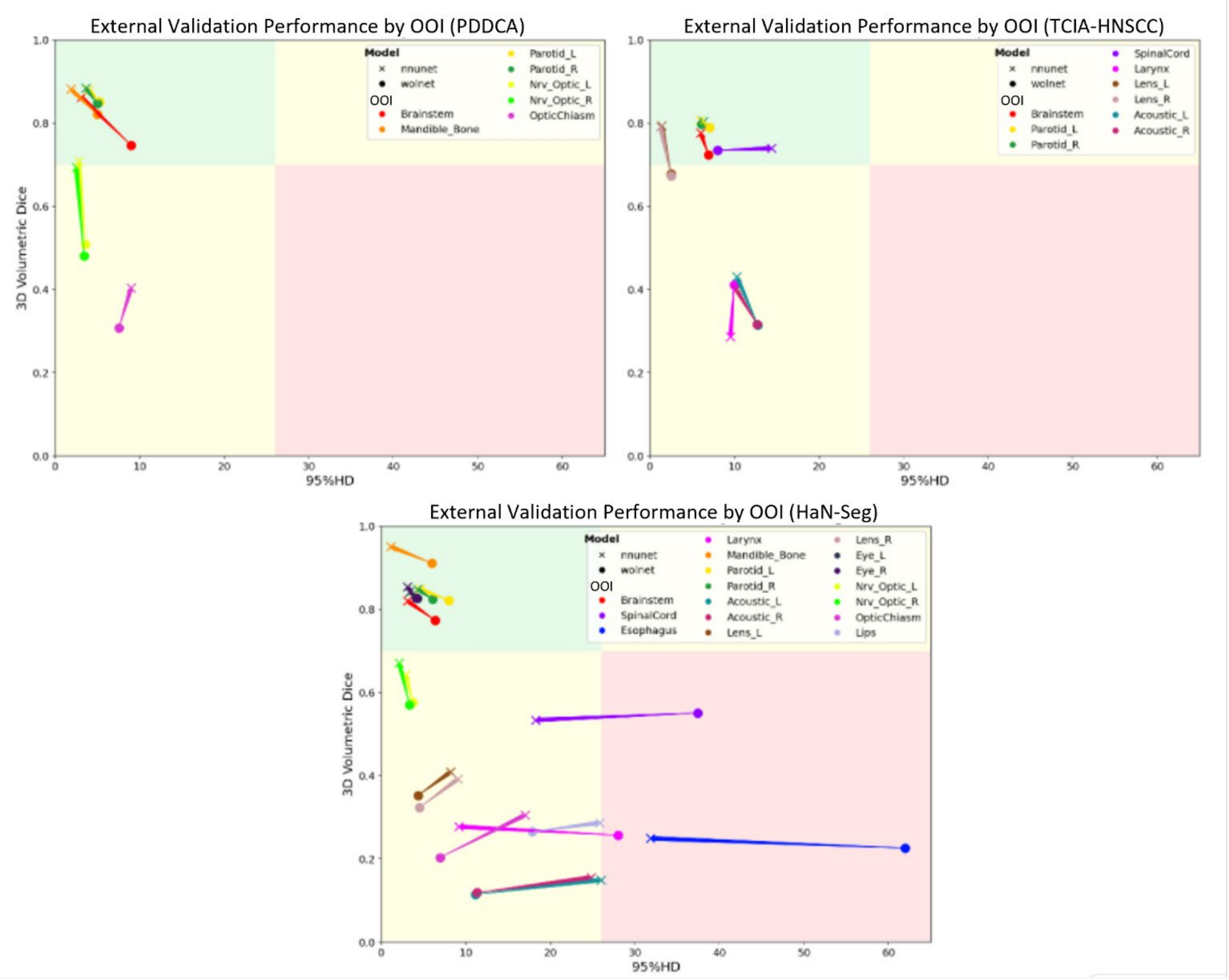

Figure S5: External Validation Performance of Fine-Tuned WOLNET vs nnUNet

This figure presents scatter plots comparing the external validation performance of two models, Fine-Tuned WOLNET and nnUNet, on various OOI (Organs of Interest) categories within the external validation datasets. The performance metrics used are the mean classical DICE coefficient (3D Volumetric Dice) and 95th percentile Hausdorff Distance (95HD). The background of the scatter plot is divided into regions based on a 30/70 split of the data ranges for the two performance metrics. The top-left region, shaded in light green, indicates the best performance (high DICE, low 95HD), while the bottom-right region, shaded in light pink, indicates the worst performance (low DICE, high 95HD).

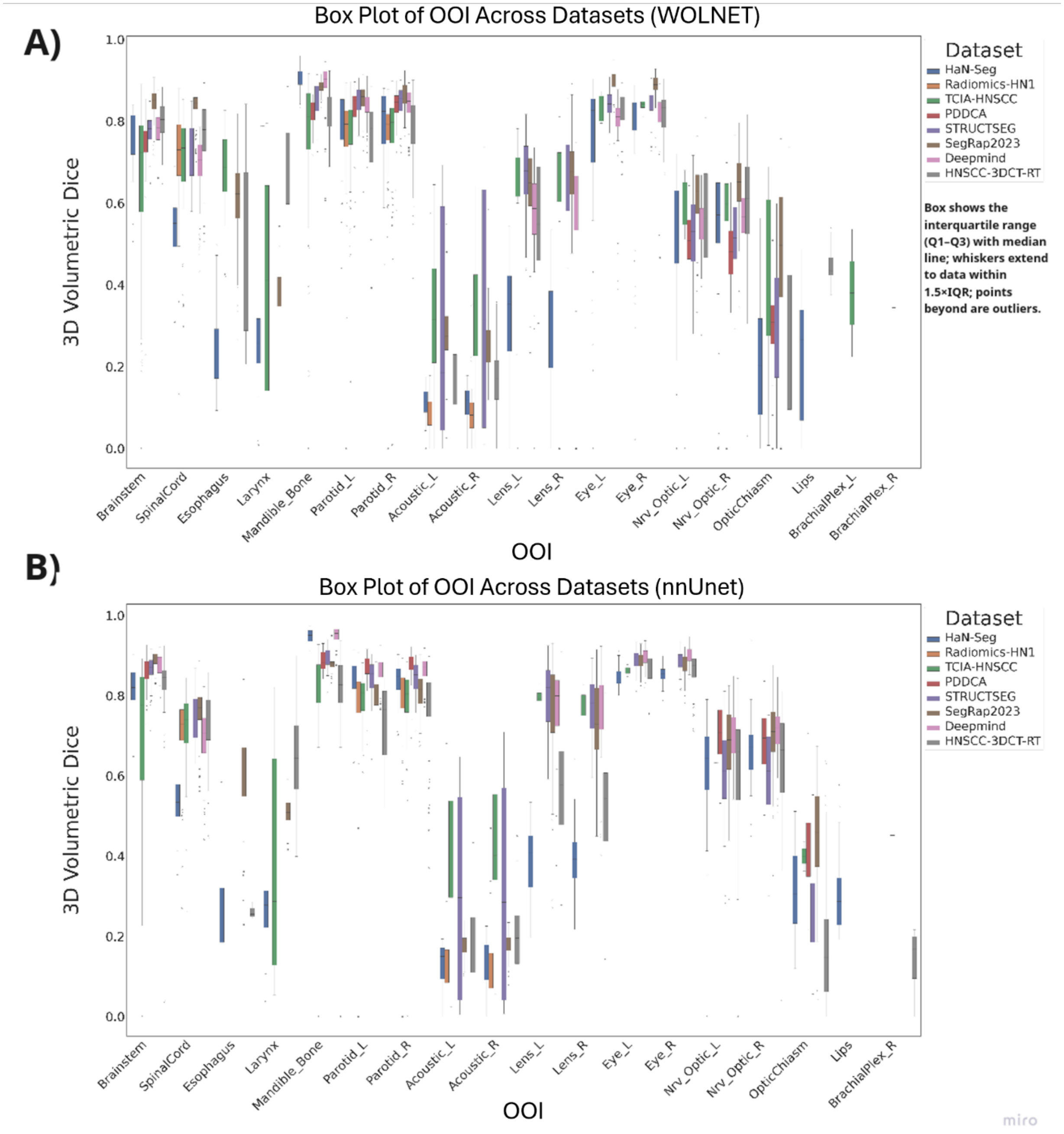

Figure S6: Generalizability Assessment of Fine-Tuned WOLNET and nnUNet. Box plots to show variation of 3D volumetric DICE of the Fine-Tuned WOLNET and nnUNet across eight external datasets. A) DICE for Fine-Tuned WOLNET, and B) DICE for nnUNet.

Discussion of Bias

Variability of Ground-Truth Contours

A source of bias in translating auto-segmentation models to clinical practice is the use of training data derived from diverse clinical environments [22]. Ground-truth contours for HNC OOI delineation generated by clinician experts exhibit inter-observer and inter-institution variability. Models trained on single-center data or single-observer data often overfit to specific institutional guidelines or observer preferences, limiting their generalizability [23–26].

To ensure broad applicability, SCARF evaluates generalizability by incorporating ground-truth contours from multiple observers and institutions. This approach mitigates institution-specific bias, providing a robust framework for benchmarking models intended for multi-center deployment [23–26]. In the case of single-center deployment, users may adapt SCARF to include ground-truth contours from the single-center at which the model will be deployed to improve specificity to the institution's guidelines.

Low-Resolution vs. High-Resolution Performance

Preliminary evaluation of all models utilized a lower resolution image set. While this greatly increases computational power and allows for longer convergence, this methodology will introduce bias for high-resolution image analysis. To limit the extent of this bias, low resolution auto-segmentations were used to select the top performing model, and that model was re-trained using the appropriate high-resolution images.

However, there will be bias introduced into the selection process. Performance accuracy for small and thin structures will be less accurate due to partial volume effects [27] and centroid approximation errors [28]. In Supplementary Figure S7, we demonstrate this bias for the WOLNET model on the RADCURE dataset. We see that performance is correlated to size of the structure, with the effect being more prevalent at low-resolution and when evaluated with DICE. The bias will be present in all models providing consistency for comparison.

CT Windowing

The ideal CT window is dependent on tissue, structure size and patient physiology [29]. In an auto-segmentation model for OOIs, the model will segment a variety of tissues that range across different ideal windows. For SCARF, to evaluate generalizability, a "universal" window was selected of [-100, 300] HU. Any window selected will introduce bias to the performance of segmentation between different structures. It is expected that high-contrast structures such as the mandible will have higher accuracy, and that larger structures will exhibit higher accuracy [4]. This is seen in the results of this study with Mandible consistently exhibiting high performance partially due to its bone-soft tissue interface and thin soft-tissue structures such as the Lips exhibiting lower performance.

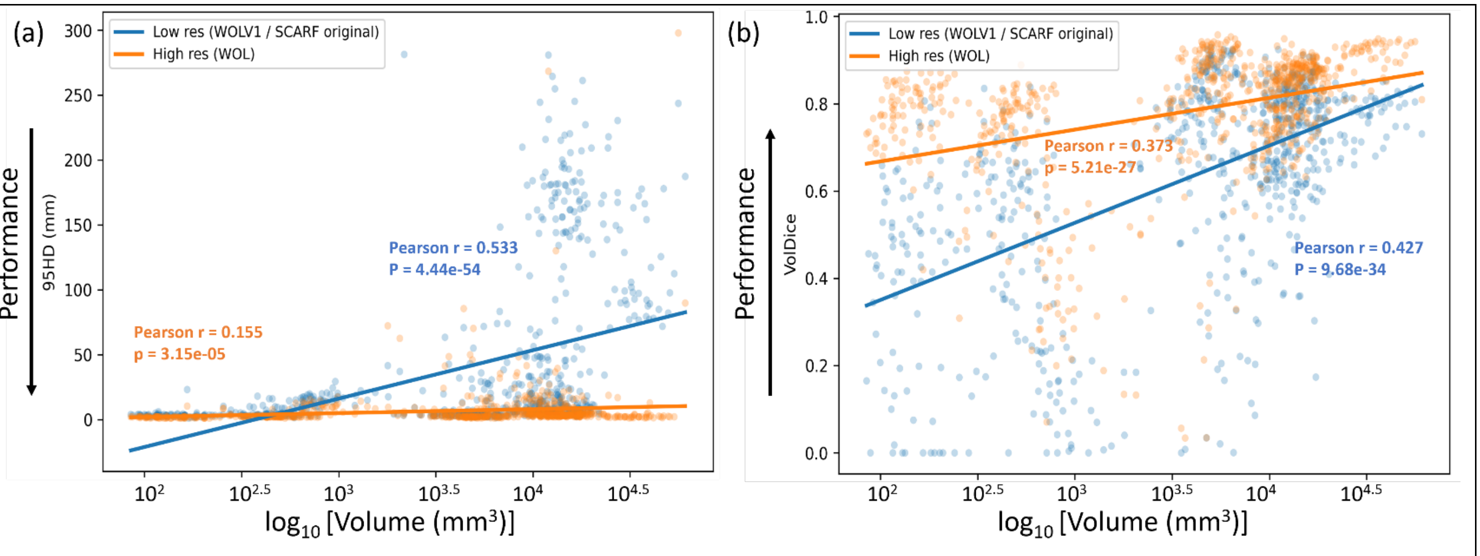

Figure S7: Correlation between anatomical structure volume and model performance of WOLNET trained with high- and low-resolution CT images for a) 95HD and b) DICE. Model performance is worse for small structures. This effect is larger with DICE metrics than with 95HD, and larger for low-resolution images.

Table S3: Median 3D Volumetric DICE Performance of Refined WOLNET

*** two contours (L/R) mandible were used as single GT mask**

**** Results shown are ensemble application without fine tuning or post processing**

OOI	RADCURE (n=59)	HNSCC-3DCT-RT (n=94)	Deepmind - Onc (n=35)	Deepmind- Rad (n=35)	PDDCA (n=48)	Radiomics-HN1 (n=129)	STRUCTSEG-19 (n=50)	TCIA-HNSCC (n=190)	SegRap2023 (n=120)	HaN-Seg (n=42)
**BSTEM**	0.87±0.04	0.80±0.05	0.77±0.05	0.80±0.04	0.75±0.09		0.78±0.04	0.72±0.18	0.85±0.03	0.77±0.07
**CHIASM**	0.38±0.11	0.27±0.21			0.31±0.10		0.34±0.15	0.55±0.24	0.5±0.18	0.2±0.15
**ESOPH**	0.82±0.07	0.56±0.23						0.69±0.12	0.62±0.1	0.23±0.11
**LACOU**	0.8±0.17	0.18±0.08				0.13±0.09	0.3±0.27	0.31±0.17	0.28±0.07	0.12±0.04
**LARYNX**	0.88±0.06	0.67±0.13						0.41±0.26	0.38±0.05	0.26±0.09
**LEYE**	0.92±0.04	0.84±0.05	0.80±0.09	0.83±0.09			0.84±0.04	0.82±0.03	0.9±0.03	0.83±0.21
**LIPS**	0.76±0.21	0.43±0.06								0.27±0.15
**LLENS**	0.79±0.06	0.55±0.15	0.57±0.07	0.62±0.1			0.68±0.09	0.68±0.18	0.65±0.1	0.35±0.15
**LOPTIC**	0.79±0.08	0.56±0.13	0.55±0.05	0.55±0.08	0.51±0.06		0.53±0.1	0.59±0.2	0.63±0.08	0.58±0.19
**LPAR**	0.87±0.04	0.78±0.10	0.84±0.04	0.84±0.04	0.85±0.07	0.79±0.09	0.86±0.04	0.79±0.09	0.86±0.04	0.82±0.09
**LPLEX**	0.73±0.13									
**MAND**	0.93±0.03	0.83±0.06	0.89±0.03	0.92±0.06	0.82±0.03		0.86±0.04*	0.81±0.16	0.89±0.02	0.91±0.03

									*	
**RACOU**	0.80±0.16	0.17±0.08				0.13±0.09	0.32±0.29	0.32±0.16	0.25±0.06	0.12±0.04
**REYE**	0.9±0.03	0.83±0.05	0.82±0.07	0.84±0.06			0.85±0.03	0.84±0.02	0.89±0.03	0.83±0.23
**RLENS**	0.82±0.07		0.58±0.09	0.62±0.09			0.68±0.10	0.67±0.21	0.68±0.09	0.32±0.16
**ROPTIC**	0.77±0.11	0.61±0.16	0.55±0.07	0.58±0.07	0.48±0.07		0.51±0.11	0.6±0.2	0.63±0.18	0.57±0.2
**RPAR**	0.86±0.03	0.81±0.09	0.85±0.04	0.86±0.04	0.85±0.06	0.80±0.09	0.86±0.04	0.8±0.09	0.87±0.03	0.82±0.09
**RPLEX**	0.73±0.14									
**SPCOR**	0.86±0.05	0.78±0.09	0.69±0.08	0.72±0.06		0.71±0.07	0.68±0.07	0.74±0.14	0.84±0.03	0.55±0.08

Table S4: Median 95HD Performance of Refined WOLNET

*** two contours (L/R) mandible were used as single GT mask**

**** Results shown are ensemble application without fine tuning or post processing**

OOI	RADCURE (n=59)	HNSCC-3DCT-RT (n=94)	Deepmind - Onc (n=35)	Deepmind- Rad (n=35)	PDDCA (n=48)	Radiomics-HN1 (n=129)	STRUCTSEG-19 (n=50)	TCIA-HNSCC (n=190)	SegRap23 (n=120)	HaN-Seg (n=42)
**BSTEM**	3.41±1.91	5.61±2.0	6.25±2.34	3.74±0.68	9.00±3.27		5.75±2.47	10.00±13.66	6.00±2.94	6.37±3.49
**CHIASM**	9.56±3.49	7.68±3.92			7.55±2.24		6.58±3.77	5.59±5.04	4.12±2.84	6.96±3.22
**ESOPH**	6.08±10.19	15.1±13.46						15.90±18.24	24.72±25.27	61.98±13.33
**LACOU**	2.18±9.48	10.04±2.14				10.20±7.62	11.14±9.84	7.27±3.11	7.81±0.95	11.15±1.06
**LARYNX**	3.08±2.44	10.20±5.41						15.72±17.71	21.00±5.01	28.00±3.74
**LEYE**	2.0±0.76	3.63±0.91	3.18±2.39	2.24±0.15			3.00±0.71	3.91±0.70	3.00±0.72	4.28±2.93
**LIPS**	4.0±10.0	14.51±4.00								17.86±11.98
**LLENS**	2.0±0.46	3.23±1.13	2.50±0.2	1.65±0.55			3.00±1.01	2.50±12.9	3.00±0.80	17.86±11.

			8					7		98
**LOPTIC**	2.0±1.13	7.09±4.80	2.80±3.09	2.24±1.88	3.59±3.96		6.43±2.35	4.08±13.36	4.24±3.70	3.72±4.96
**LPAR**	4.0±3.65	8.46±4.36	5.03±55.25	3.74±5.17	5.28±2.61	7.81±31.70	4.78±2.47	9.65±5.46	5.83±3.29	8.00±5.26
**LPLEX**	5.67±13.51							49.34±26.10		
**MAND**	2.0±39.97	7.25±8.20	2.50±182.95	1.00±0.42	5.00±15.60		11.48±19.82*	13.00±58.19	6.63±1.71	6.00±4.23
**RACOU**	2.18±9.67	10.56±53.95				10.45±22.50	10.01±9.71	7.16±3.03	7.81±0.95	11.36±1.32
**REYE**	2.0±0.75	4.00±0.88	2.24±0.14	3.07±1.74			3.00±0.53	3.76±0.43	3.00±0.59	4.12±3.31
**RLENS**	1.84±0.52		1.95±0.31	2.50±0.29			3.00±0.75	2.50±13.16	3.00±0.61	4.53±1.86
**ROPTIC**	2.0±1.78	6.66±4.95	2.24±0.42	2.84±1.36	3.43±1.34		6.66±2.83	3.28±13.81	3.00±2.46	3.35±7.22
**RPAR**	4.0±34.43	6.96±4.76	3.16±2.36	5.00±113.68	5.00±2.85	7.28±18.15	5.43±3.65	9.39±6.39	3.51±3.68	6.08±4.62
**RPLEX**	6.0±14.91							58.26±0		
**SPCOR**	8.31±18.52	4.00±7.39	3.16±1.27	5.65±26.47		27.90±11.99	3.78±2.57	19.99±49.14	3.61±10.62	37.41±47.34

Table S5: Median 3D Volumetric DICE Performance of nnUNet

**OOI**	**HaN-Seg (n=42)**	**RADCURE (n=59)**	**Deep mind - Onc (n=35)**	**Deep mind- Rad (n=35)**	**Radiomics -HN1 (n=129)**	**TCIA-HNSCC (n=190)**	**HNSCC-3DCT-RT (n=94)**	**PDDCA (n=48)**	**SegRap23 (n=120)**	**STRUCT SEG-19 (n=50)**
**BSTEM**	0.82±0.06	0.92±0.03	0.88±0.03	0.89±0.02		0.78±0.21	0.85±0.15	0.86±0.04	0.89±0.02	0.87±0.04
**CHIASM**	0.31±0.10	0.75±0.14				0.40±0.05	0.15±0.16	0.40±0.10	0.46±0.12	0.23±0.11

**ESOPH**	0.25±0.11	0.87±0.07				0.67	0.26±0.02		0.61±0.10	
**LACOU**	0.15±0.05	0.87±0.16			0.12±0.10	0.43±0.18	0.19±0.09		0.18±0.03	0.30±0.25
**LARYNX**	0.28±0.08	0.90±0.07			0.79	0.29±0.29	0.64±0.13		0.51±0.03	
**LEYE**	0.84±0.03	0.94±0.03	0.89±0.02	0.91±0.02		0.86±0.02	0.87±0.04		0.89±0.02	0.90±0.03
**LIPS**	0.29±0.09	0.81±0.09								
**LLENS**	0.41±0.08	0.87±0.08	0.79±0.07	0.83±0.11		0.79±0.02	0.58±0.17		0.80±0.12	0.82±0.08
**LOPTIC**	0.64±0.14	0.84±0.07	0.72±0.06	0.72±0.07		0.63	0.66±0.17	0.71±0.07	0.69±0.09	0.61±0.12
**LPAR**	0.85±0.05	0.90±0.04	0.87±0.03	0.87±0.04	0.80±0.10	0.81±0.06	0.77±0.10	0.88±0.05	0.81±0.04	0.86±0.05
**LPLEX**		0.79±0.09								
**MAND**	0.95±0.02	0.96±0.02	0.95±0.02	0.96±0.01		0.85±0.15	0.83±0.17	0.88±0.03	0.88±0.01	0.89±0.03
**RACOU**	0.16±0.05	0.86±0.16			0.11±0.08	0.40±0.17	0.20±0.09		0.18±0.03	0.28±0.27
**REYE**	0.85±0.02	0.94±0.02	0.90±0.02	0.91±0.02		0.86	0.87±0.05		0.88±0.03	0.89±0.02
**RLENS**	0.39±0.09	0.87±0.08	0.77±0.10	0.80±0.10		0.79±0.04	0.54±0.17		0.73±0.10	0.78±0.08
**ROPTIC**	0.67±0.08	0.83±0.08	0.72±0.07	0.72±0.06		0.72	0.67±0.16	0.69±0.08	0.71±0.08	0.61±0.10
**RPAR**	0.85±0.04	0.90±0.04	0.87±0.03	0.87±0.03	0.82±0.10	0.80±0.11	0.80±0.10	0.88±0.04	0.82±0.05	0.85±0.04
**RPLEX**		0.78±0.09				0.45	0.17±0.07			
**SPCOR**	0.53±0.06	0.88±0.04	0.68±0.09	0.74±0.07	0.73±0.09	0.74±0.17	0.74±0.07		0.77±0.05	0.74±0.06

Table S6: Median 95HD Performance of nnUNet

OOI	HaN-Seg (n=42)	RADCURE (n=59)	Deepmind - Onc (n=35)	Deepmind- Rad (n=35)	Radiomics-HN1 (n=129)	TCIA-HNSCC (n=190)	HNSCC-3DCT-RT (n=94)	PDDCA (n=48)	SegRap23 (n=120)	STRUCTSEG-19 (n=50)
**BSTEM**	3.11±1.62	2.00±1.34	2.94±0.63	2.77±0.66		6.00±6.33	4.12±15.45	3.10±0.77	1.83±0.77	3.21±1.12
**CHIASM**	17.05±7.17	2.24±1.63				8.73±1.53	12.42±6.16	9.01±2.39	9.12±5.00	12.71±3.68
**ESOPH**	31.83±10.01	4.06±10.69				8	28.02±1.28		25.52±16.91	

**LACOU**	26.01±9.55	2.00±7.74			21.86±23.04	10.29±5.37	16.00±4.36		39.00±5.20	21.28±20.37
**LARYNX**	9.19±2.26	2.24±2.89			4.47	9.53±16.25	7.71±3.38		11.49±4.15	
**LEYE**	3.33±0.83	1.41±0.49	1.96±0.41	1.96±0.35		2.69±0.26	2.83±0.86		1.10±0.25	1.56±0.58
**LIPS**	25.85±10.48	2.64±1.38								
**LLENS**	8.19±4.71	1.00±0.53	1.55±0.71	1.25±1.21		1.39±0.37	2.24±0.84		1.13±3.04	1.17±0.83
**LOPTIC**	2.92±3.81	2.00±0.95	2.50±11.82	2.50±12.49		20.2	6.41±7.72	2.82±1.76	24.32±17.52	15.94±5.13
**LPAR**	4.58±59.51	2.83±2.37	5.00±71.68	4.93±71.59	5.80±3.93	5.96±5.22	7.00±7.50	3.99±61.18	6.35±3.03	4.13±2.35
**LPLEX**		5.06±8.53								
**MAND**	1.12±0.62	1.41±19.77	0.98±53.79	0.98±53.78		5.00±5.70	4.00±5.00	1.88±0.48	1.87±0.60	3.05±1.48
**RACOU**	24.82±9.21	2.00±7.81			27.01±42.28	10.20±5.00	14.21±4.15		38.85±4.88	19.40±20.67
**REYE**	3.12±0.81	1.41±0.48	1.96±0.38	1.95±0.36		2.83	2.24±0.86		1.10±0.55	1.63±0.63
**RLENS**	9.10±4.63	1.00±0.60	2.50±0.68	1.28±0.91		1.24±0.63	2.24±1.16		1.82±2.97	1.45±0.84
**ROPTIC**	2.13±3.09	2.00±1.77	2.50±4.10	2.26±5.03		16.6	11.00±7.13	2.50±1.52	21.37±23.64	16.14±5.25
**RPAR**	4.29±2.76	3.00±2.86	4.00±66.28	4.68±65.94	5.95±52.26	6.34±5.75	7.04±6.05	3.63±2.30	6.10±4.18	4.04±3.50
**RPLEX**		5.09±9.19				83.5	202.21±38.36			
**SPCOR**	18.27±21.13	4.00±9.13	14.34±13.03	14.19±13.20	15.29±7.67	14.40±22.01	12.45±10.37		12.03±5.25	9.61±6.26

Table S7: Median 3D Volumetric DICE Performance of Selected Models

**OOI**	**ANA**	**DEN**	**HIGH**	**PIP**	**RES**	**RSA**	**TIR**	**U++**	**U3P**	**VNET**
**BSTEM**	0.81±0.06	0.62±0.13	0.80±0.06	0.80±0.05	0.59±0.09	0.43±0.12	0.27±0.06	0.76±0.06	0.59±0.12	0.75±0.08
**CHIASM**	0.23±0.12	0.00±0.11	0.32±0.14	0.24±0.13	0.02±0.08			0.07±0.10	0.21±0.13	0.03±0.09
**ESOPH**			0.46±0.13	0.58±0.13			0.29±0.13	0.55±0.13	0.12±0.11	0.42±0.16
**LACOU**	0.71±0.14	0.57±0.16	0.72±0.15	0.68±0.14	0.61±0.13	0.64±0.13		0.72±0.15	0.69±0.15	0.64±0.12
**LARYNX**	0.85±0.09	0.76±0.11	0.77±0.07	0.78±0.08	0.70±0.11	0.69±0.12	0.46±0.08	0.77±0.13	0.72±0.14	0.81±0.09
**LEYE**	0.80±0.06	0.59±0.11	0.85±0.04	0.68±0.07		0.00±0.02		0.59±0.18	0.69±0.09	0.78±0.05
**LIPS**	0.68±0.20	0.54±0.19	0.68±0.19	0.67±0.19	0.57±0.20	0.39±0.17	0.44±0.14	0.68±0.19	0.66±0.19	0.53±0.19
**LLENS**	0.43±0.18	0.00±0.16		0.45±0.17			0.04±0.02	0.24±0.20	0.18±0.18	0.00±0.13

**LOPTIC**	0.33±0.18			0.38±0.16	0.02±0.04			0.42±0.16	0.55±0.14	0.00±0.03
**LPAR**	0.66±0.11	0.43±0.14	0.65±0.13	0.71±0.09	0.54±0.14	0.55±0.14	0.69±0.08	0.72±0.11	0.67±0.11	0.76±0.07
**LPLEX**	0.60±0.09	0.50±0.09	0.36±0.10	0.50±0.11	0.33±0.09	0.03±0.03	0.00±0.01	0.54±0.10	0.41±0.09	0.55±0.10
**MAND**	0.55±0.08	0.69±0.10	0.55±0.06	0.62±0.09	0.58±0.06	0.60±0.07	0.02±0.01	0.53±0.07	0.84±0.05	0.48±0.06
**RACOU**	0.69±0.14	0.50±0.16	0.74±0.15	0.70±0.16	0.58±0.13	0.60±0.14		0.70±0.15	0.71±0.16	0.73±0.15
**REYE**	0.78±0.07	0.61±0.11	0.84±0.04	0.68±0.08		0.00±0.02		0.61±0.17	0.76±0.09	0.80±0.07
**RLENS**	0.42±0.15	0.00±0.13		0.40±0.14			0.03±0.01	0.35±0.20	0.17±0.20	0.00±0.06
**ROPTIC**	0.43±0.19			0.39±0.15	0.06±0.09			0.48±0.22	0.50±0.19	0.00±0.02
**RPAR**	0.67±0.10	0.53±0.14	0.61±0.13	0.74±0.08	0.61±0.10	0.46±0.12	0.64±0.13	0.75±0.10	0.72±0.08	0.74±0.06
**RPLEX**	0.59±0.11	0.46±0.09	0.36±0.10	0.48±0.11	0.34±0.10	0.03±0.03	0.32±0.07	0.57±0.10	0.44±0.11	0.51±0.10
**SPCOR**	0.76±0.05	0.77±0.05	0.79±0.05	0.76±0.05	0.73±0.06	0.70±0.06	0.67±0.07	0.78±0.04	0.71±0.04	0.78±0.04

Table S8: Median 95HD Performance of Selected Model

**OOI**	**ANA**	**DEN**	**HIGH**	**PIP**	**RES**	**RSA**	**TIR**	**U++**	**U3P**	**VNET**
**BSTEM**	6.78± 38.54	15.52± 47.32	92.09± 67.61	38.91± 55.05	15.56± 47.75	27.64± 32.91	255.75± 23.19	7.44± 14.96	10.20± 44.35	8.49± 16.75
**CHIASM**	14.41± 3.22	15.63± 4.64	12.00± 42.53	13.78± 2.75	16.76± 3.64		23.20± 6.07	17.75± 3.40	14.92± 3.76	17.22± 3.40
**ESOPH**			35.16± 54.02	13.65± 35.69			27.37± 39.76	22.11± 37.27	59.06± 32.48	24.80± 19.11
**LACOU**	3.32± 11.54	4.85± 12.16	3.00± 27.47	3.32± 11.82	4.24± 11.90	4.12± 37.33	285.06± 29.49	3.16± 27.49	3.16± 18.27	3.87± 19.10
**LARYNX**	3.61± 41.72	6.71± 47.23	174.78± 26.80	173.07± 48.35	118.89± 36.47	83.28± 47.50	133.85± 47.92	177.11± 59.57	188.00± 81.82	5.83± 51.61
**LEYE**	3.61± 4.18	6.16± 10.14	3.16± 0.49	5.39± 1.95		17.26± 1.84	28.25± 2.61	6.08± 39.16	4.24± 1.23	4.24± 1.30
**LIPS**	5.00± 17.27	7.87± 22.56	66.63± 113.37	6.00± 78.80	6.00± 65.45	10.86± 35.39	58.29± 44.24	222.87± 125.74	108.26± 79.71	32.26± 80.17
**LLENS**	3.16± 29.94	3.46± 1.37		3.61± 1.34			161.39± 74.87	3.45± 1.28	3.62± 0.84	4.12± 1.10
**LOPTIC**	7.21± 6.94			9.80± 7.35	19.41± 8.56		29.56± 2.22	5.79± 26.18	4.25± 3.18	21.54± 5.69
**LPAR**	126.23± 53.41	179.91± 39.62	181.85± 40.90	162.31± 32.88	130.25± 61.65	18.68± 21.24	12.21± 4.20	161.56± 42.06	161.14± 27.52	12.53± 57.84
**LPLEX**	31.52± 25.81	44.22± 46.35	137.47± 20.08	122.61± 40.88	123.07± 42.75	64.52± 34.44	62.14± 24.40	105.80± 40.40	34.13± 36.31	111.10± 33.88
**MAND**	133.35± 34.95	50.25± 62.12	167.13± 39.20	191.95± 34.52	166.02± 23.13	126.94± 15.69	235.71± 25.02	107.88± 37.23	87.67± 64.39	164.37± 29.92
**RACOU**	3.61± 11.26	5.83± 11.77	3.00± 11.39	3.16± 60.38	5.00± 12.02	4.24± 11.94	287.16± 29.05	3.06± 11.31	3.29± 11.15	3.16± 11.91
**REYE**	3.74± 44.48	6.00± 1.94	3.16± 0.55	5.00± 1.19		17.98± 2.72	26.34± 2.10	5.83± 81.64	3.61± 1.16	3.61± 1.22

**RLENS**	3.23± 0.71	4.68± 1.86		3.61± 1.33			175.44± 59.47	3.42± 1.08	3.74± 1.16	5.10± 0.93
**ROPTIC**	5.74± 4.81			7.89± 5.61	19.24± 5.65			7.04± 6.86	4.47± 4.64	18.40± 5.31
**RPAR**	125.47± 52.22	194.55± 31.89	187.49± 32.71	154.77± 51.18	111.14± 57.23	24.08± 28.12	11.69±4.44	161.44± 54.34	154.18± 48.09	15.81± 79.56
**RPLEX**	27.73± 37.97	44.65± 46.73	136.15± 17.85	108.15± 58.24	112.37± 41.40	94.94± 33.66	30.41±23.25	108.71± 43.74	32.93± 27.75	121.56± 39.73
**SPCOR**	48.00± 34.72	33.00± 25.30	51.24± 34.75	52.84± 28.20	102.02± 31.38	47.42± 31.99	57.59± 47.62	40.02± 31.76	42.14± 37.56	17.86± 25.51

Table S5: SCARF's toolkit can be used to facilitate compliance to checklists like that provided by CONSORT-AI

**SCARF Step**		**SCARF Tool**	**Facilitates CONSORT-AI Compliance**		**Time Savings (Dev. Hours)**
**Data Curation**	1	**MedImg-Tools package allows for consistent processing of internal/external datasets**	Allows standardization and processing of data, can be used to facilitate compliance to section:	5.ii - 5.iv	240
**Model Curation**	2	Suite of open-source CNN's modified to train 3D auto-segmentation models using pyTorch lightning. Weights of best model used in Clinical Acceptability and generalizability assessment provided	Systematic approach to model versioning and training supervised auto-segmentation modes proposed	5.i, 5.v	80
**Model Training**	3	Streamlined PyTorch Lightning boilerplate allows for easy model integration, training & inference in less than 10 lines of code			
**Performance Assessment**	4	Collection of easy to use scripts/notebooks that makes performance assessment of model easy	[5.v] Assessment of contours generated for 19 OOI(s) used in radiation therapy planning of HNC. Quantitative metrics can be associated with qualitative metrics to discuss harms/limitations of method when segmenting specific organs of interest	5.v, 19	10

**Clinical Assessment**	5	**Quannotate platform enables web-based blinded clinical assessment of contours**	Clinical assessment of contours allows experts to rate quality of predictions, and can be used for bias assessment (How would the expert rating change when seeded with the name of contour generator?)	5.vi, 19-20	100
**Method Generalizability**	6	Collection of scripts and notebooks that makes generalizability assessment (inference) on external datasets using both cpus/gpus easy	Collection and processing of 6 external datasets allows for external validity of best model performance, can be used to assess similarities/discrepancies of contouring methods used across centers	21-22	20

References

[1] Liu L, Jiang H, He P, et al. On the Variance of the Adaptive Learning Rate and Beyond 2021. https://doi.org/10.48550/arXiv.1908.03265.

[2] Liu L, Jiang H, He P, et al. On the Variance of the Adaptive Learning Rate and Beyond. Proceedings of the Eighth International Conference on Learning Representations (ICLR 2020), 2020.

[3] Beare R, Lowekamp B, Yaniv Z. Image Segmentation, Registration and Characterization in *R* with **SimpleITK**. J Stat Soft 2018;86. https://doi.org/10.18637/jss.v086.i08.

[4] Bibb R, Eggbeer D, Paterson A. Medical imaging. Medical Modelling. 2nd ed., Woodhead Publishing; 2015. https://doi.org/10.1016/B978-1-78242-300-3.00002-0.

[5] Cardoso MJ, Li W, Brown R, et al. MONAI: Medical Open Network for AI 2022. https://doi.org/10.48550/arXiv.2211.02701.

[6] Kiser KJ, Barman A, Stieb S, et al. Novel Autosegmentation Spatial Similarity Metrics Capture the Time Required to Correct Segmentations Better Than Traditional Metrics in a Thoracic Cavity Segmentation Workflow. J Digit Imaging 2021;34:541–53. https://doi.org/10.1007/s10278-021-00460-3.

[7] Çiçek Ö, Abdulkadir A, Lienkamp SS, et al. 3D U-Net: Learning Dense Volumetric Segmentation from Sparse Annotation. In: Ourselin S, Joskowicz L, Sabuncu MR, et al., editors. Medical Image Computing and Computer-Assisted Intervention – MICCAI 2016, Cham: Springer International Publishing; 2016, p. 424–32. https://doi.org/10.1007/978-3-319-46723-8_49

[8] Wolny A, Cerrone L, Vijayan A, et al. Accurate and versatile 3D segmentation of plant tissues at cellular resolution. eLife 2020;9:e57613. https://doi.org/10.7554/eLife.57613.

[9] Lee K, Zung J, Li P, et al. Superhuman Accuracy on the SNEMI3D Connectomics Challenge 2017. https://doi.org/10.48550/arXiv.1706.00120.

[10] Li W, Wang G, Fidon L, et al. On the Compactness, Efficiency, and Representation of 3D Convolutional Networks: Brain Parcellation as a Pretext Task. In: Niethammer M, Styner M, Aylward S, et al., editors. Information Processing in Medical Imaging, Cham: Springer International Publishing; 2017, p. 348–60. https://doi.org/10.1007/978-3-319-59050-9_28

[11] Nikolaos A. Deep learning in medical image analysis: a comparative analysis of multi-modal brain-MRI segmentation with 3D deep neural networks. Master’s Thesis. University of Patras, 2019.
[12] Fang X, Yan P. Multi-Organ Segmentation Over Partially Labeled Datasets With Multi-Scale Feature Abstraction. IEEE Trans Med Imaging 2020;39:3619–29. https://doi.org/10.1109/TMI.2020.3001036.
[13] Huang H, Lin L, Tong R, et al. UNet 3+: A Full-Scale Connected UNet for Medical Image Segmentation. ICASSP 2020 - 2020 IEEE International Conference on Acoustics, Speech and Signal Processing (ICASSP), 2020, p. 1055–9. https://doi.org/10.1109/ICASSP40776.2020.9053405.
[14] Zhou Z, Rahman Siddiquee MM, Tajbakhsh N, et al. UNet++: A Nested U-Net Architecture for Medical Image Segmentation. In: Stoyanov D, Taylor Z, Carneiro G, et al., editors. Deep Learning in Medical Image Analysis and Multimodal Learning for Clinical Decision Support, vol. 11045, Cham: Springer International Publishing; 2018, p. 3–11. https://doi.org/10.1007/978-3-030-00889-5_1.
[15] Zhu W, Huang Y, Zeng L, et al. AnatomyNet: Deep learning for fast and fully automated whole-volume segmentation of head and neck anatomy. Medical Physics 2019;46:576–89. https://doi.org/https://doi.org/10.1002/mp.13300.
[16] Yu L, Cheng J-Z, Dou Q, et al. Automatic 3D Cardiovascular MR Segmentation with Densely-Connected Volumetric ConvNets. In: Descoteaux M, Maier-Hein L, Franz A, et al., editors. Medical Image Computing and Computer-Assisted Intervention − MICCAI 2017, Cham: Springer International Publishing; 2017, p. 287–95. https://doi.org/10.1007/978-3-319-66185-8_33
[17] Jegou S, Drozdzal M, Vazquez D, et al. The One Hundred Layers Tiramisu: Fully Convolutional DenseNets for Semantic Segmentation. 2017 IEEE Conference on Computer Vision and Pattern Recognition Workshops (CVPRW), Los Alamitos, CA, USA: IEEE Computer Society; 2017, p. 1175–83. https://doi.org/10.1109/CVPRW.2017.156.
[18] Zhang H, Zhang J, Zhang Q, et al. RSANet: Recurrent Slice-Wise Attention Network for Multiple Sclerosis Lesion Segmentation. In: Shen D, Liu T, Peters TM, et al., editors. Medical Image Computing and Computer Assisted Intervention – MICCAI 2019, Cham: Springer International Publishing; 2019, p. 411–9. https://doi.org/10.1007/978-3-030-32248-9_46
[19] Milletari F, Navab N, Ahmadi S-A. V-Net: Fully Convolutional Neural Networks for Volumetric Medical Image Segmentation. 2016 Fourth International Conference on 3D Vision (3DV), 2016, p. 565–71. https://doi.org/10.1109/3DV.2016.79.
[20] Ravi N, Gabeur V, Hu Y-T, et al. SAM 2: Segment Anything in Images and Videos 2024. https://doi.org/10.48550/arXiv.2408.00714.
[21] Ma J, Yang Z, Kim S, et al. MedSAM2: Segment Anything in 3D Medical Images and Videos 2025. https://doi.org/10.48550/arXiv.2504.03600.
[22] Welch ML, Grant B, Deutschman C, et al. A practical framework for operationalising responsible and equitable artificial intelligence in health care: tackling bias, inequity, and implementation challenges. The Lancet Digital Health 2026:100957. https://doi.org/10.1016/j.landig.2025.100957.
[23] Cardenas CE, Yang J, Anderson BM, et al. Advances in Auto-Segmentation. Seminars in Radiation Oncology n.d.;29:185–97. https://doi.org/10.1016/j.semradonc.2019.02.001.
[24] Pang EPP, Tan HQ, Wang F, et al. Multicentre evaluation of deep learning CT autosegmentation of the head and neck region for radiotherapy. Npj Digit Med 2025;8:312. https://doi.org/10.1038/s41746-025-01624-z.
[25] Saeed N, Hassan S, Hardan S, et al. A Multimodal and Multi-centric Head and Neck Cancer Dataset for Segmentation, Diagnosis and Outcome Prediction 2025. https://doi.org/10.48550/arXiv.2509.00367.

[26] Van Dijk LV, Van Den Bosch L, Aljabar P, et al. Improving automatic delineation for head and neck organs at risk by Deep Learning Contouring. Radiotherapy and Oncology 2020;142:115–23. https://doi.org/10.1016/j.radonc.2019.09.022.
[27] Monnin P, Sfameni N, Gianoli A, et al. Optimal slice thickness for object detection with longitudinal partial volume effects in computed tomography. J Applied Clin Med Phys 2017;18:251–9. https://doi.org/10.1002/acm2.12005.
[28] Horner M, Luke SM, Genc KO, et al. Towards Estimating the Uncertainty Associated with Three-Dimensional Geometry Reconstructed from Medical Image Data. J Verif Valid Uncertain Quantif 2019;4:041002. https://doi.org/10.1115/1.4045487.
[29] Huo Y, Tang Y, Chen Y, et al. Stochastic tissue window normalization of deep learning on computed tomography. J Med Imag 2019;6:1. https://doi.org/10.1117/1.JMI.6.4.044005.
